# Voxel Design of Grayscale DLP 3D‐Printed Soft Robots

**DOI:** 10.1002/advs.202309932

**Published:** 2024-05-20

**Authors:** Mengjie Zhang, Xiru Fan, Le Dong, Chengru Jiang, Oliver Weeger, Kun Zhou, Dong Wang

**Affiliations:** ^1^ State Key Laboratory of Mechanical System and Vibration School of Mechanical Engineering Shanghai Jiao Tong University Shanghai 200240 China; ^2^ Meta Robotics Institute Shanghai Jiao Tong University Shanghai 200240 China; ^3^ Cyber‐Physical Simulation Group & Additive Manufacturing Center Department of Mechanical Engineering Technical University of Darmstadt Dolivostr. 15, Darmstadt 64293 Hessen Germany; ^4^ Singapore Centre for 3D Printing School of Mechanical and Aerospace Engineering Nanyang Technological University Singapore 639798 Singapore

**Keywords:** grayscale DLP 3D printing, hyperelastic constitutive model, multimodal soft robots, programmable mechanical behaviors, voxel‐based finite‐element model

## Abstract

Grayscale digital light processing (DLP) printing is a simple yet effective way to realize the variation of material properties by tuning the grayscale value. However, there is a lack of available design methods for grayscale DLP 3D‐printed structures due to the complexities arising from the voxel‐level grayscale distribution, nonlinear material properties, and intricate structures. Inspired by the dexterous motions of natural organisms, a design and fabrication framework for grayscale DLP‐printed soft robots is developed by combining a grayscale‐dependent hyperelastic constitutive model and a voxel‐based finite‐element model. The constitutive model establishes the relationship between the projected grayscale value and the nonlinear mechanical properties, while the voxel‐based finite‐element model enables fast and efficient calculation of the mechanical performances with arbitrarily distributed material properties. A multiphysics modeling and experimental method is developed to validate the homogenization assumption of the degree of conversion (DoC) variation in a single voxel. The design framework is used to design structures with reduced stress concentration and programmable multimodal motions. This work paves the way for integrated design and fabrication of functional structures using grayscale DLP 3D printing.

## Graphical abstract

A design and fabrication framework for grayscale DLP printing is developed by combining a grayscale‐dependent hyperelastic constitutive model and a voxel‐based finite‐element model. A multiphysics modeling and experimental method is developed to validate the homogenization assumption of the degree of conversion variation of a single voxel. The framework is used in reducing stress concentration and multimodal soft robots.

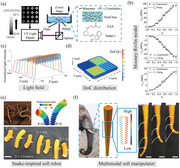

## 1 Introduction

3D printing, or additive manufacturing, has gained increasing attention due to its ability to fabricate intricate structures with high throughput and resolution. Such nature renders it applicable in soft robots,^[^ ^1^, ^2^, ^3^, ^4^, ^5^ ^]^ bioengineering,^[^ ^6^, ^7^, ^8^ ^]^ active functional materials,^[^ ^9^, ^10^, ^11^ ^]^ and optical devices.^[^ ^12^, ^13^ ^]^ However, traditional 3D printing methods mainly focus on objects with uniform material property. Nevertheless, anisotropic materials are ubiquitous in biological systems and exhibit excellent functionalities, such as the differential swelling of spruce branches,^[^ ^14^ ^]^ shear force reduction of the multimaterial sucker rings in squids,^[^ ^15^ ^]^ and force endurance of the hierarchical myotendinous junctions.^[^ ^16^ ^]^ The ability to precisely assign multiple material properties during 3D printing is therefore desired to enhance functionality.

Recent years have witnessed several 3D printing methods that can realize multiple material properties. Examples include the multinozzle fused deposition modeling,^[^ ^17^, ^18^, ^19^ ^]^ direct ink writing,^[^ ^20^, ^21^, ^22^, ^23^ ^]^ multimaterial PolyJet printing,^[^ ^24^, ^25^, ^26^ ^]^ multimaterial stereolithography, and grayscale digital light processing (DLP) methods.^[^ ^27^, ^28^, ^29^, ^30^, ^31^, ^32^ ^]^ Among them, the grayscale DLP 3D printing can offer precise material property distribution, strong bonding, high freedom in structural design, and high fabrication efficiency. High accuracy is achievable as the resolution is the size of one pixel in the projected 2D images. By tuning the light intensity of each pixel, precise material property distribution can be realized. The interfacial bonding is strong as covalent crosslinking is formed. Moreover, complex structures with cavities can be fabricated without additional supporting materials using the bottom‐up DLP 3D printing. The photocuring resin solidifies one layer at a time without material shifting and removal, reducing the printing time considerably.

Grayscale DLP 3D printing has been widely used to fabricate multifunctional structures because of the differences in material properties and functionalities caused by the nonuniform light doses. For example, Kuang et al. designed functionally graded metamaterials, 4D‐printed structures, and diffusion‐assisted coloring using different modulus, glassy transition temperatures, and diffusivity caused by the grayscale value, respectively.^[^ ^33^ ^]^ Grayscale DLP printing has also been used to control the local stimuli‐responsive performances of hydrogel.^[^ ^34^, ^35^ ^]^ To reduce the trial and error and labor in experiments, researchers recently have proposed several models for grayscale DLP 3D printing to improve the printing accuracy and predict deformation. The printing accuracy is mainly determined by the degree of conversion (DoC) inside the printed structures. A methodology that combines the Gaussian beam representation of light and a reaction–diffusion model was developed to fabricate subpixel features by manipulating the pixel‐level grayscale (G) value.^[^ ^36^, ^37^, ^38^ ^]^ Emami et al. incorporated the Gaussian beam convergence and divergence and studied their effects on the DoC distribution in grayscale DLP printing.^[^ ^39^ ^]^ Due to the large design space provided by the grayscale distribution, optimization methods were also developed to inversely design the grayscale distribution based on multiphysics models to improve the printing accuracy and resolution.^[^ ^40^, ^41^ ^]^

The anisotropic modulus caused by the different grayscale values induces diverse deformation modes without changing the geometries. By virtue of the residual stress generated during grayscale frontal photopolymerization, 3D origami structures have been designed based on a beam model. Combining the DoC distribution and finite‐element (FE) simulations, a model was developed to predict the 4D programmable shape change induced by the nonuniform internal stress.^[^ ^42^ ^]^ Considering the nonlinear behaviors of the grayscale‐printed materials, Valizadeh et al. modeled the mechanical properties of grayscale‐printed blocks using a hyperelastic constitutive equation.^[^ ^43^ ^]^

However, the above models only investigated simple structures, as predicting the deformed shapes of complex structures leads to complexities such as voxel‐level grayscale distribution, DoC variation in a single voxel, complex structures, and nonlinear material properties. As the grayscale values can be accurately assigned on the voxel level, the progress in modeling is hindered due to the tremendous design space and multimaterial distributions. DoC varies inside a single voxel because of phenomena such as Gaussian light distribution and light absorption during the printing process. The printed materials are generally soft and exhibit finite deformation, with nonlinear dependence on the grayscale value. The complex geometries enabled by DLP 3D printing further complicate the modeling.

In this work, we develop a voxel design and fabrication framework for the grayscale DLP printing of structures by integrating a grayscale‐dependent hyperelastic model and a voxel‐based FE model. The grayscale‐dependent hyperelastic constitutive model is developed to establish the relationship between the projected grayscale value and the nonlinear mechanical properties. The voxel‐based FE model predicts the structures' mechanical behaviors with arbitrarily distributed materials fast and efficiently. We ignore DoC variation due to the significantly increased computation cost and treated a single voxel as a homogenized one whose mechanical properties depend only on its G value. To validate the above assumption, a method combining multiphysics modeling and experiments is developed. The DoC distributions under single‐ and multi‐photopolymerization are calculated, and results show that DoC distributions within a voxel volume are similar among voxels with the same G and differ significantly among voxels with different G, which validates the homogenization assumption. We use a hybrid resin that enables high stretchability (>60%) and strong recovery of the printed structures. The developed design and fabrication framework is used to reduce the stress concentration and design multimodal locomotion soft robots. Quantitative comparisons are conducted to validate the methods. The printing accuracy is characterized. The mesh and voxel quality and sensitivity are evaluated. The developed design and manufacturing framework allows for predicting mechanical behaviors quantitatively according to the grayscale distributions and provides a way for the inverse design of the grayscale distribution.

## 2 Results

### 2.1 Grayscale DLP 3D Printing

**Figure 1:**
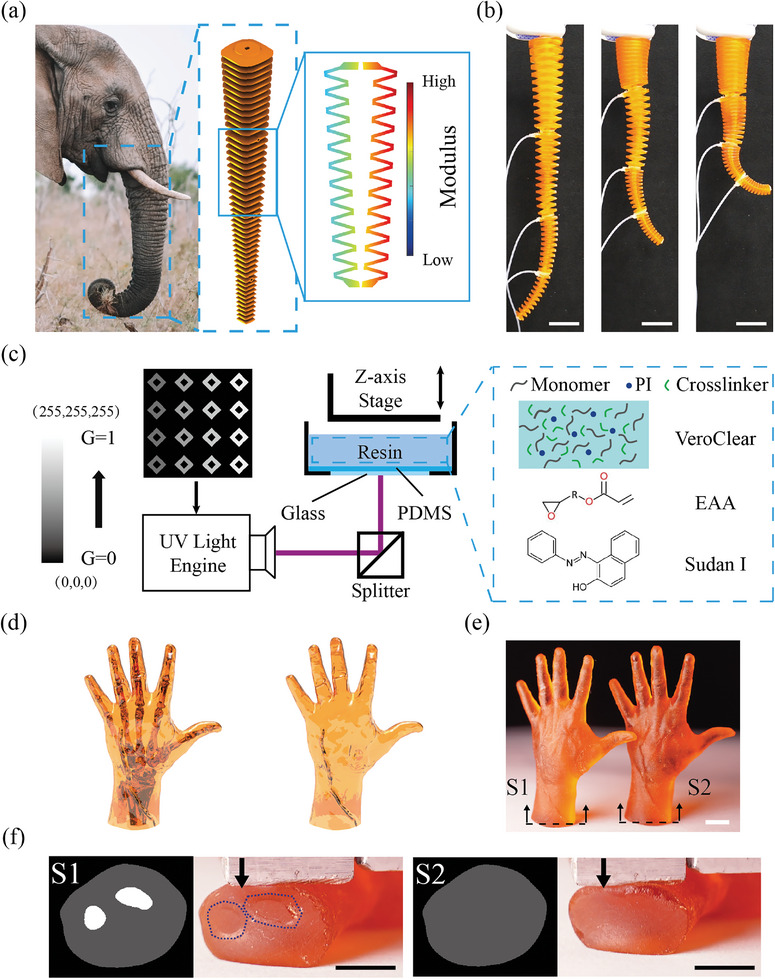
Grayscale DLP printing. a) Inspired by the dexterous motion of an elephant trunk. b) Multimodal soft robots designed and fabricated using grayscale DLP 3D printing. c) Schematic illustration of the grayscale DLP printing with hybrid resin VEAA. G = 1 represents (255,255,255) and G = 0 represents (0,0,0). d) CAD model of human hands with bones and without bones. e) Grayscale‐printed human hands with different G distributions. f) Slice images of the cross section of two hands S1 and S2 and optical images of the indentation experiments. G = 1 for the bright part (stiff bone) and G = 0.2 for the gray part (soft tissue) (Scale bar: 5 mm).

Natural organisms exhibit multimodal dexterous motions. For example, elephant trunks can form complex trajectories such as bending, twisting, and elongating because of the muscle fiber distributions (**Figure** **1**a).^[^ ^44^ ^]^ Inspired by the material anisotropy generated by the muscle fiber distributions, we fabricate and design multimodal soft manipulators using grayscale DLP 3D printing (Figure 1b). The grayscale DLP printing system composed of a UV‐projector (DLi 3DLP9000, including a 405 nm UV light source and a DMD module), a beam splitter, a resin tank with a transparent glass window coated with polydimethylsiloxane (PDMS) membrane, and a linear translation stage. The hybrid resin (hereinafter referred to as VEAA) was prepared by adding epoxy aliphatic acrylate (EAA) to the commercial UV‐curable resin VeroClear together with Sudan I as the photo‐absorber (Figure 1c). Before printing, the CAD model was sliced into images with desired layer thickness. The grayscale value for each pixel was assigned according to the desired material distributions using MATLAB code. The grayscale patterns were then projected to cure the resin to form structures layer by layer. The size of each voxel is about 42 µm × 42 µm × 20 µm.

The grayscale values determine the projected UV light intensity on each pixel, which affects the DoC and the mechanical properties of the cured layers. Here, we define a parameter G to describe the grayscale value. G = 1 represents full light intensity with RGB(255,255,255), and G = 0 means nearly zero light intensity. We measured the light intensity as G ranging from 0 to 1 in Figure S1a. The relationship between the light intensity and G is almost linear.

Using different G distributions, we demonstrate grayscale‐printed human hands with and without bones. The CAD models of human hands with bones and without bones are shown in Figure 1d. Different G distributions are illustrated in the slice images. In the slice images in Figure 1f, the bright part was assigned with G = 1, representing the stiff bone of the printed human hand due to the large light intensity. The gray part used G = 0.2 to imitate the soft tissues of the printed hand. For comparison, a uniform value of G = 0.2 was used for the hand without bones. Details such as the veins can be observed from the snapshots of the two printed hands in Figure 1e, showing a high printing precision. While it is difficult to distinguish the two hands from their appearances, indentation experiments in Figure 1f clearly show the stiffness differences. The experimental snapshot of the structures showed an apparent bone contour (blue dashed lines), while the structures without bones exhibited a uniform deformation. The relatively smaller deformation in the human hand with bones also indicates that it can withstand larger compression forces. Besides tuning the material properties, grayscale values can also be used to tune the printing quality (Figure S4).

### 2.2 Voxel Finite Deformation Model

A voxel finite deformation model was developed to predict the large deformation of grayscale DLP 3D‐printed structures. The nonlinear behaviors of materials printed under various grayscale values G were characterized by a grayscale‐dependent Mooney–Rivlin hyperelastic constitutive model. A series of dogbone samples (Figure S1b) were printed with G varying from 0.05 to 1 to obtain the dependence of the material properties on G. The stress–strain curves of the printed samples under uniaxial loading are shown in Figure S1c. The dependences of Young's moduli *E* and fracture strain on G obtained from the stress–strain curves are shown in Figure S1d. Young's modulus *E* (fitted at 3% strain) increased nonlinearly from ≈3 MPa to ≈ 200 MPa as G increased from 0.05 to 1, spanning two orders of magnitude. The sharp increase of *E* fell in the range when G increased from 0.3 and 0.7. The apparent increase in the modulus resulted from the increasing crosslinking density.

The fracture strain increased with G first and then decreased slightly. The sample was overpolymerized when G is larger than 0.85, which decreased the fracture strain. The largest fracture strain was nearly 70% at G ≃ 0.8, which was almost seven times of VeroClear's fracture strain.^[^ ^45^ ^]^ During UV‐triggered photopolymerization, the EAA monomers formed extended linear chains compared to those in the original network of VeroClear. The length increase in the linear chains greatly improved the stretchability of the network system.^[^ ^46^ ^]^ The significant increase in stretchability enables the fabrication of complex structures with large deformations.

From the stress–strain curves shown in Figure S1c, the material exhibited nonlinear mechanical behaviors and finite deformation. We developed a grayscale‐dependent Mooney–Rivlin hyperelastic constitutive model to capture the material nonlinearity. The three‐parameter Mooney–Rivlin model was chosen with the strain energy density function *W* as

(1) $$W=C_{10}\left(I_{1}-3\right)+C_{01}\left(I_{2}-3\right)+C_{11}\left(I_{1}-3\right)\;\left(I_{2}-3\right),$$

where *C*_10_, *C*_01_, and *C*_11_ are the three grayscale‐dependent material constants. The parameters *I*_1_ and *I*_2_ are the invariants of the right Cauchy–Green strain tensor **C** = **F** ^*T*^ **F** defined as follows:

(2) $$\begin{array}{c} I_{1}=tr\left(C\right)=\lambda_{1}^{2}+\lambda_{2}^{2}+\lambda_{3}^{2}\\ I_{2}=\frac{1}{2}\left[{\left(tr(C)\right)}^{2}-tr\left(C^{2}\right)\right]=\lambda_{1}^{2}\lambda_{2}^{2}+\lambda_{1}^{2}\lambda_{3}^{2}+\lambda_{2}^{2}\lambda_{3}^{2} \end{array}$$

with principal stretches λ_*i*_, where *i* = 1, 2, 3. The matrix **F** is the deformation gradient. The materials are assumed to be incompressible, and *λ*_1_ *λ*_2_ *λ*_3_ = 1. The principal Cauchy stress with respect to the corresponding stretch ratio^[^ ^47^ ^]^ is

(3) $$\sigma_{i}=\lambda_{i}\frac{\partial W}{\partial \lambda_{i}}=\lambda_{i}\left(\frac{\partial W}{\partial I_{1}}\frac{\partial I_{1}}{\partial \lambda_{i}}+\frac{\partial W}{\partial I_{2}}\frac{\partial I_{2}}{\partial \lambda_{i}}\right).$$

Hence,

(4) $$\begin{array}{ccc} \sigma_{1}-\sigma_{3} & = & 2C_{10}\left(\lambda_{1}^{2}-\lambda_{3}^{2}\right)\\ & & +2C_{11}\left(\lambda_{1}^{2}-\lambda_{3}^{2}\right)\left(\frac{1}{\lambda_{1}^{2}}+\frac{1}{\lambda_{2}^{2}}+\frac{1}{\lambda_{3}^{2}}-3\right)\\ & & -2C_{01}\left(\frac{1}{\lambda_{1}^{2}}-\frac{1}{\lambda_{3}^{2}}\right)-2C_{11}\left(\frac{1}{\lambda_{1}^{2}}-\frac{1}{\lambda_{3}^{2}}\right)\\ & & \times (\lambda_{1}^{2}+\lambda_{2}^{2}+\lambda_{3}^{2}-3). \end{array}$$

For a simple uniaxial tensile test, $\lambda_{1}=\lambda ,\lambda_{2}=\lambda_{3}=1/\sqrt{\lambda }$, and *σ*_3_ = 0. The engineering stress $\sigma_{1}^{\mathrm{Eng}}=\sigma_{1}/\lambda_{1}$. Therefore, the relationship between $\sigma_{1}^{\mathrm{Eng}}$ and *λ*_1_ can be obtained as

(5) $$\begin{array}{ccc} \sigma_{1}^{\mathrm{Eng}} & = & 2C_{10}\left(\lambda -\frac{1}{\lambda^{2}}\right)+2C_{01}\left(1-\frac{1}{\lambda^{3}}\right)\\ & & +6C_{11}\left(\lambda -\frac{1}{\lambda^{2}}\right)\left(\lambda -1-\frac{1}{\lambda }+\frac{1}{\lambda^{2}}\right). \end{array}$$

**Figure 2:**
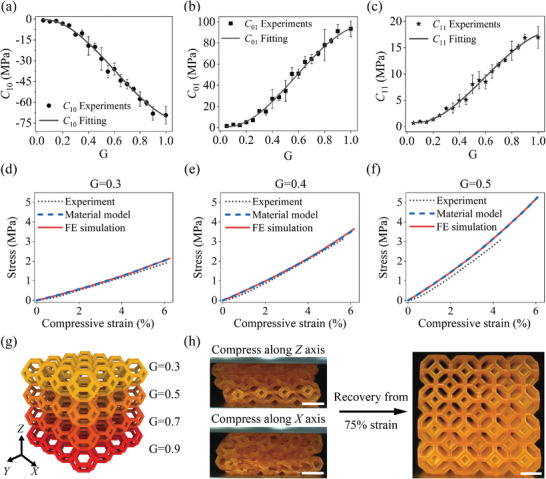
Grayscale‐dependent hyperelastic model. a–c) Dependences of the material constants *C*_10_, *C*_01_, and *C*_11_ of the grayscale‐printed dogbone samples on G. d–f) Comparison between the experimental, theoretical, and FE‐simulated stress–strain curves of the uniaxial compression tests of grayscale‐printed cylinders. g) CAD model of the functionally graded Kelvin foam structure with G varying from 0.3 to 0.9. h) The snapshots of the printed Kelvin foam's recovery from 75% compression strain along *Z*‐axis and *X*‐axis, respectively.

By fitting Equation (5) with the stress–stretch ratio curves of the grayscale‐printed dogbone samples under different G values from 0.1 to 1, the material constants *C*_10_, *C*_01_, and *C*_11_ were obtained. The comparisons between the experimental and theoretically fitted curves are shown in Figure S3. The theoretically fitted *C*_10_, *C*_01_, and *C*_11_ and the corresponding adjusted *R* ^2^ values are shown in Table S1. The adjusted *R* ^2^ are all above 0.9, indicating the three‐parameter grayscale‐dependent Mooney–Rivlin model can well capture the behaviors of the grayscale‐printed samples. The dependence of *C*_10_, *C*_01_, and *C*_11_ on G are shown in **Figure** **2**a–c. Note that *C*_10_ is negative, while *C*_01_ and *C*_11_ are positive. All the magnitudes of *C*_10_, *C*_01_, and *C*_11_ increase with G. Third‐order polynomial functions (solid curves) are fitted to predict the material parameters $C_{10}\left(G\right)$, $C_{01}\left(G\right)$, and $C_{11}\left(G\right)$ with arbitrary G, given as

(6) $$\begin{array}{c} C_{10}\left(G\right)=114.3G^{3}-207.3G^{2}+23.99G-1.143\\ C_{01}\left(G\right)=-167.1G^{3}+291.9G^{2}-32.98G+2.011\\ C_{11}\left(G\right)=-29.7G^{3}+53.34G^{2}-7.168G+0.987 \end{array}.$$

A python‐assisted FE method correlating the nonlinear material property with the voxel grayscale pattern was developed to predict the mechanical behaviors. FE simulations were conducted in Abaqus 2020 (Dassault Systems, Waltham, MA, USA). Home‐written python scripts were used in FE simulation to assign material properties at a voxel scale (Section S5 in Supporting Information).

Uniaxial compression tests were conducted in accordance with the ISO 7743 standards to validate the model, employing the Type A test procedure. Grayscale‐printed standard cylinders with a design diameter of 29 mm and height of 12.5 mm were tested. The surfaces of the cylinder were lubricated by silicone oil. Specimens were printed using G = 0.3, 0.4, and 0.5 with a layer thickness of 20 µm. The measured diameters and heights were 28.9 and 12.5 mm for samples with G = 0.3, 28.8 and 12.6 mm for G = 0.4, and 28.8 and 12.6 mm for G = 0.5. The apparent height difference resulted from the increasing thickness of the initially printed layers.

Figure S2a and b illustrates the experimental and FE simulation setups. In FE simulations, the penalty formulation was chosen, and the friction coefficient was set at 0.001. The comparison of the experimental, FE‐simulated, and theoretical stress–strain curves are shown in Figure 2d–f. The theoretical result was obtained through Equation (5). The experimental curves exhibited a slight discrepancy, lower than the FE‐simulated curves. This deviation may be attributed to the lower modulus of the thick initial layers, where the thicker layer led to a reduction in modulus.

The ability to tune the materials' stiffness using G distributions was then validated by grayscale DLP 3D‐printed functionally graded materials. A functionally graded 4 × 4 × 4 Kelvin foam structure was printed with gradient G as shown in Figure 2g. The illustration of the slice image is shown in Figure S2c. The color in the CAD model and the grayscale slice image show the G distribution. The graded direction of G was along the *Z*‐axis. Four G values were used for each layer of unit cell: G = 0.3, 0.5, 0.7, and 0.9. The snapshot of the printed samples shows good printing quality. Uniaxial compression tests were conducted along the graded direction (along the *Z*‐axis) and the nongraded direction (along the *X*‐axis) (Figure S2d, Movie S1). Sequential deformation could be observed upon compression along the graded direction. The softest layer (G = 0.4) was squeezed first, followed by the deformation of stiffer layers with higher G. When compressed along the nongraded direction, each layer showed a similar axial strain.

As shown in Figure S2e, the experimental instantaneous modulus increased with the axial strain when compressed in the graded direction because of the graded G value (solid black line). In contrast, the stress increased linearly with the strain initially, followed by a slight decrease and another sharp increase upon compression in the nongraded direction (red dash line). The slight decrease was due to the buckling of the structures, while the sharp increase resulted from the contact of different layers. Both structures showed full recoveries after a 75% compression strain in a short time (Figure 2h), which is promising in highly stretchable devices. FE simulations were conducted for quantitative comparison, which agreed well with the experimental results. The plateau was observed at ≈37% strain because of the overall buckling upon compression along the *X*‐axis. Note that the Abaqus simulations abort due to the severe distortion resulted from contact or overall buckling.

### 2.3 Characterization of Printing Accuracy

**Figure 3:**
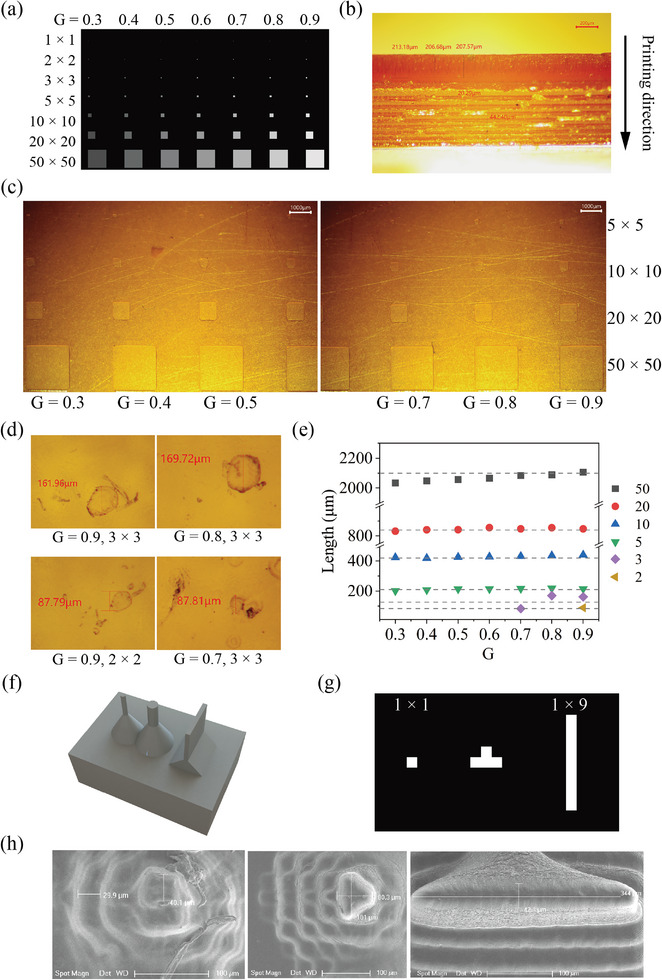
Dimensions characterization of grayscale‐printed structures. a) Grayscale square patterns for input. b) Microscopy image of a typical printed rectangle structure. The measured thicknesses of the initial and later‐printed layers are 209 ± 2.81 and 20.1 ± 0.2 µm, respectively. c and d) The microscopy images of the printed squares. e) Comparison of the measured (markers) and designed (dashed lines) lengths. f) CAD models for trapezoid structures featuring a single pixel. g) Slice image of the top surface of the models. h) SEM images of the trapezoid structures, revealing successful printing of all single‐pixel features.

Experiments were undertaken to assess the dimensional accuracy of the grayscale‐printed structures. **Figure** **3**a shows the grayscale patterns input to the projector, featuring squares ranging from 1 pixel × 1 pixel to 50 pixels × 50 pixels, with G ranging from 0.3 to 0.9. An MSD9224 stereomicroscope (Murzider, China) was used to observe and measure the printed features.

Figure 3b shows the lateral microscopy image of a typical printed rectangular structure. Distinctions between layers of the later printed layers could not be observed, while apparent differences existed for the initially printed layers. The measured thickness of the initial layers was 209 ± 2.81 µm, contributing to the overall increased height. The increase in the layer thickness might result from the deformation of the membrane or variations in the initial height of the printing platform. As the light dose for each layer remained constant, the modulus of the initial layers was relatively small, leading to lower experimental stress. The thickness of the layer printed later was 20.1 ± 0.2 µm, agreeing well with the design thickness (20 µm). The layer thickness error was within 1.5% in the *Z* direction.

Twenty layers shown in (a) were printed on the substrate, and the resulting microscopy images are shown in Figure 3c and d. Structures with dimensions of 5 pixels × 5 pixels or larger were successfully printed. Squares of 3 pixels × 3 pixels could only be printed using G ⩾ 0.7, and those of 2 pixels × 2 pixels were observed exclusively under G = 0.9. The 1 pixel × 1 pixel squares were not observed. Figure 3e compares the measured (markers) and designed (dashed lines) lengths. While the sizes of structures with dimensions of 5 pixels × 5 pixels or above align well with the designed size, 3 pixels × 3 pixels structures exhibit a notable discrepancy. Detailed characterizations of the printing quality with square patterns are given in Table S2.

Printing an isolated 1‐pixel structure is challenging, primarily because of the limited contact areas with the substrate. To address it, we have designed trapezoid structures with increasing contact areas with the substrate, as illustrated in Figure 3f. Slice images of the top surfaces are presented in Figure 3g. All three designs featured a single pixel. Scanning electron microscopy (SEM) images of the printed structures are displayed in Figure 3h, revealing the successful printing of all single‐pixel features with maximum errors within 5% of the featured dimension. However, some distortion existed, attributed to light propagation. Based on the above experiments, the minimum feature structures should be equal to or larger than 5 pixels.

### 2.4 DoC Distributions

The DoC varies inside a single voxel due to many phenomena during the printing process, such as light scattering, radial diffusion, Gaussian light distribution, and light absorption. In the theoretical model, we ignored DoC variation because of the significantly increased computation cost and treated a single voxel as a homogenized one whose mechanical properties depend solely on its G value. This homogenization procedure is based on the following two assumptions:

(1) The DoC distributions within a voxel volume are similar among voxels with the same G.
(2) The DoC distributions differ significantly among voxels with different G.

To ensure the accuracy of voxel design, we developed a method combining multiphysics modeling and experiments. This method allows us to calculate the DoC distribution within the structures and validate the two aforementioned assumptions. The Beer–Lambert law and Gaussian beam propagation are considered. Light scattering is negligible as the resin is optically clear before and after curing. The diffusion of various chemical species is also ignored as the printing process is fast.^[^ ^48^ ^]^

**Figure 4:**
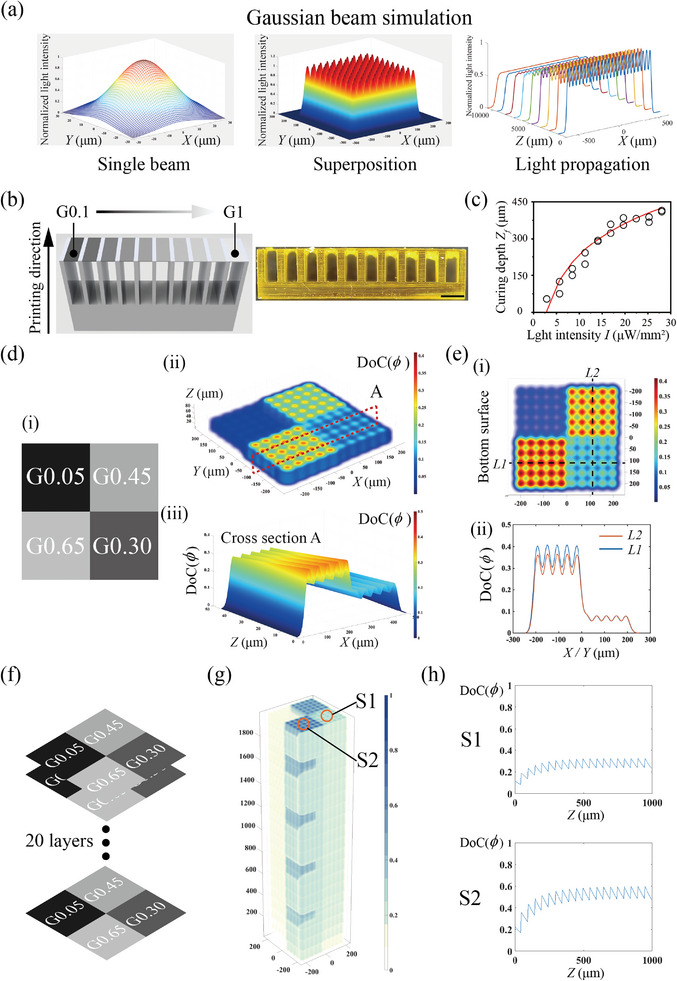
DoC distribution. a) Light intensity of i) a single Gaussian beam, ii) multiple Gaussian beams, and iii) the propagation of multiple Gaussian beams. b) CAD model and printed structures for measuring the curing depth under different G from 0.1 to 1. c) Dependence of curing depth on light intensity under various UV exposure time. d) Grayscale pattern and calculated DoC distribution. e) Calculated DoC distribution of the bottom surface along the *X* (*L*1) and *Y* (*L*2) axis. f) Grayscale patterns and g) corresponding DoC distribution during multilayer photopolymerization. h) Dependence of DoC on the *Z* direction of the two columns at positions S1 and S2.

The light intensity distribution projected from each pixel is assumed to follow the Gaussian distribution (**Figure** **4**a), which could be written on the focal plane (*z* = 0) as

(7) $$I\left(x,y,z=0\right)=I_{0}\mathrm{Exp}\left[\frac{-2\left({\left(x-x_{0}\right)}^{2}+{\left(y-y_{0}\right)}^{2}\right)}{{\omega_{0}}^{2}}\right],$$

where *I*_0_ is the peak light intensity in the center (*x*_0_, *y*_0_). The parameter *ω*_0_ refers to the waist radius of the Gaussian beam, which is equal to the radius when the light intensity reaches *I*_0_/*e* ^2^, where *e* is the Euler's number. The light field is the superposition of Gaussian beams from each pixel. The normalized light intensity of 10 pixels × 10 pixels is shown in the middle part of Figure 4a.

As the Gaussian beam propagates, the light intensity still follows the Gaussian distribution while the beam waist *ω*(*z*) along the *Z*‐axis becomes

(8) $$\omega \left(z\right)=\omega_{0}\sqrt{1+{\left(z/z_{R}\right)}^{2}},$$

where *z*_*R*_ = π*ω*_0_^2^/*λ* is the Rayleigh range with *λ* being the beam wavelength.^[^ ^39^ ^]^ Assuming there is zero energy lost during propagation, the light intensity distribution beyond the focal plane can be obtained as

(9) $$I\left(x,y,z\right)=I_{0}\left[\frac{1}{1+{\left(\frac{z}{z_{R}}\right)}^{2}}\right]\mathrm{Exp}\left[\frac{-2\left({\left(x-x_{0}\right)}^{2}+{\left(y-y_{0}\right)}^{2}\right)}{{\omega_{0}}^{2}\left[1+{\left(\frac{z}{z_{R}}\right)}^{2}\right]}\right].$$

The right side of Figure 4a shows the light propagation. Here, *ω*_0_ is 30 µm, and the size of a single pixel is 42 µm × 42 µm, which is measured from the light field of the UV projector. The light absorption follows the Beer–Lambert Law

(10) $$\frac{\partial I(r,z,t)}{\partial z}=-\mu_{0}I\left(r,z,t\right).$$

By assuming that the resin is photo‐invariant, *μ*_0_ is the attenuation coefficient. The rate change of of DoC *ϕ*(*r*, *z*, *t*) is proportional to the light intensity and uncured resin, which can be written as

(11) $$\frac{\partial \phi (r,z,t)}{\partial t}=K\left[1-\phi \left(r,z,t\right)\right]I\left(r,z,t\right),$$

where *K* is the overall reaction conversion rate. The curing depth *Z*_*f*_ can be calculated by combining Equations (10) and (11) as

(12) $$Z_{f}=\frac{1}{\mu_{0}}\ln\left[KI_{0}t/\ln\left(\frac{1}{1-\phi_{c}}\right)\right],$$

where *ϕ*_*c*_ is critical conversion.

Cantilever beam structures were printed using different G to obtain the parameters *μ*_0_ and *K*. The CAD design and printed sample are shown in Figure 4b. The thickness of the top layer is the curing depth *Z*_*f*_. *μ*_0_ and *K* were obtained to be 5.4 mm^−1^ and 0.042 ${\text{mm}}^{2}\mu W^{-1}$ by fitting Equation (12) and the measured curing depth (Figure 4c).

Under multilayer photopolymerization, the previously printed layer were further cured by the penetrated light when printing sequential layers. The reaction conversion during the *n* ^th^ light radiation in layer‐by‐layer printing can be obtained from Equation (11) as

(13) $$\phi_{n}=1+\left(\phi_{n-1}-1\right)\exp\left(-K\int I_{n}\left(r,z,t\right)dt\right),$$

where *I*_*n*_ is the intensity of the *n* ^th^ light radiation. The DoC under multiexposure can be recursively calculated by

(14) $$\begin{array}{ccc} \phi_{n}\left(r,z,t\right) & = & 1-\exp\left[-K\int_{\Delta t}I_{1}\left(r,z,t\right)dt\right.\\ & & \left.-K\int_{\Delta t}I_{2}\left(r,z,t\right)dt\text{…}\;\text{…}-K\int_{\Delta t}I_{n}\left(r,z,t\right)dt\right]. \end{array}$$

We then used the theoretical model to calculate the DoC distributions under single and multilayer photopolymerization. Figure 4d shows the DoC distribution under single‐layer photopolymerization. A rectangle of 10 pixels × 10 pixels was printed using four different G values (0.05, 0.45, 0.65, and 0.30) as shown in Figure 4d‐i. Each grayscale value covered an area with 5 pixels × 5 pixels. The 3D distribution of DoC is shown in Figure 4d‐ii. The DoC distribution of cross section A is shown in Figure 4d‐iii. Figure 4e‐i shows the DoC at the bottom surface. The DoC along lines *L*_1_ and *L*_2_ is plotted in Figure 4e‐ii. The DoC distribution within a voxel volume is similar among voxels with the same G on the *XY* plane, while it varies significantly for voxels with different G.

Note that there is a gradient zone between regions with different G values. The light field of a prescribed grayscale distribution composed of four parts with different G is modeled. The grayscale pattern is shown in Figure S5a. Figure S5b shows the 3D plot of the normalized light intensity. We plot the light distribution on lines *I*1 and *I*2 in Figure S5c. A gradient zone between regions with different G values can be observed. The gradient zone is defined as the interval between the valley and the peak of two adjacent waves with different G. The width of the gradient zone is estimated as ≈20 µm.

We then study the DoC distribution in the *Z* direction under multilayer photopolymerization. 20 layers with the same grayscale pattern were printed. The layer thickness was set at 100 µm. The grayscale pattern is given in Figure 4f, and the DoC distribution is shown in Figure 4g. The DoC distributions at positions S1 and S2 along the *Z* axis are plotted in Figure 4h. The DoC inside a voxel was similar along the *Z* direction, except for the last three to four cured layers. Therefore, the two assumptions are validated, and it is reasonable to treat a single voxel as a homogenized one whose mechanical properties depend solely on its G value.

### 2.5 Mesh and Voxel Quality Evaluation and Sensitivity

**Figure 5:**
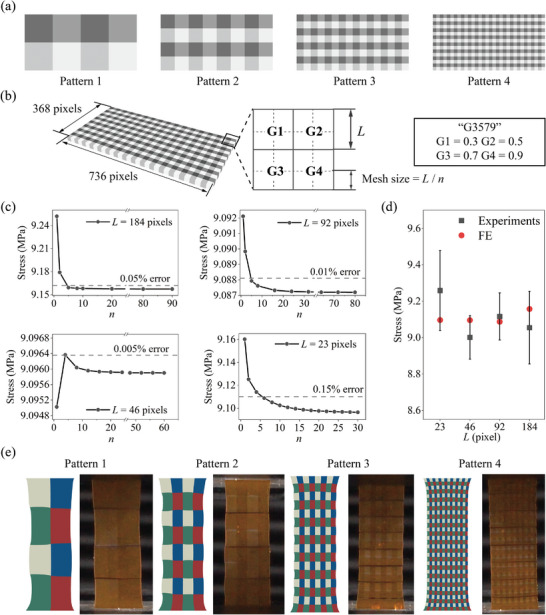
Effects of mesh and voxel sizes. a) Four periodically arranged patterns. b) Dimensions and grayscale value distributions (G3579) of the cuboid structure. c) Mesh convergence analysis for grayscale‐printed cuboid structures with four different patterns. d) Quantitative comparisons between the experimental and converged FE‐simulated stresses. e) Comparison of the experimental and FE‐simulated deformed shapes.

Mesh and voxel quality evaluation and sensitivity of grayscale‐printed structures were explored by combining experiments and FE simulations. Four different patterns were used, as illustrated in **Figure** **5**a. A cuboid with dimensions of 368 pixels × 736 pixels was used. Figure 5b shows the cuboid with four patterns. The periodic unit of each pattern comprised four squares with varying grayscale distributions (G1, G2, G3, and G4). The side lengths *L* of each square consisted of 184 pixels, 92 pixels, 46 pixels, and 23 pixels for the four different patterns, respectively. The size of each pixel was 42 µm × 42 µm. To assess the mesh convergence, various mesh sizes were chosen in the FE simulations by dividing *L* by an integer factor *n*, starting from 1. CPS8R mesh elements were used.

Figure 5c shows the mesh convergence analysis for the grayscale‐printed cuboids with four different patterns. The G distributions G1 = 0.3, G2 = 0.5, G3 = 0.7, and G4 = 0.9 (denoted as G3579) were used. Uniaxial tensile tests were simulated with an applied strain of 37.5%. The calculated stress applied to both ends of the cuboid is plotted against the mesh sizes. The results indicate rapid convergence of the calculated stress with *n*, reaching within 0.15% errors at *n* = 5. Experiments were conducted to validate the FE‐simulated results. The slice images and experimental snapshots are shown in Figure S6a and b. Quantitative comparisons between the experimental and converged FE‐simulated stresses for the four patterns are presented in Figure 5d, demonstrating reasonable agreement. Figure 5e compares the experimental and FE‐simulated deformed shapes. The deformed shapes align well, with more uniform overall deformation observed at smaller *L*.

Mesh and voxel sensitivity were also conducted for other G distributions G1 = 0.3, G2 = 0.8, G3 = 0.8, and G4 = 0.3 (denoted as G3883), as illustrated in Figure S7, yielding similar conclusions. The convergence in pattern 4 for G3883 was much slower due to the small voxel size. In this work, *n* is set to be greater than or equal to 5 for mesh size to ensure that errors remain below 0.15%. Although a finer mesh can reduce computational error, it also increases computational costs.

Voxel quality is characterized in Figure S8, showing the microscopy images of the G3579 samples. The measured *L* was around 0.933, 1.882, 3.784, and 7.530 mm for patterns 1–4, respectively. The size errors fell within 3.4%. Quantitative results are given in Table S3.

## 3 Voxel Design

The grayscale‐dependent Mooney–Rivlin model was used to program the mechanical performance of grayscale‐printed structures. Reducing the stress concentration of a plate with a central circular hole and designing multimodal pneumatic soft robots are demonstrated.

### 3.1 Reducing Stress Concentration

**Figure 6:**
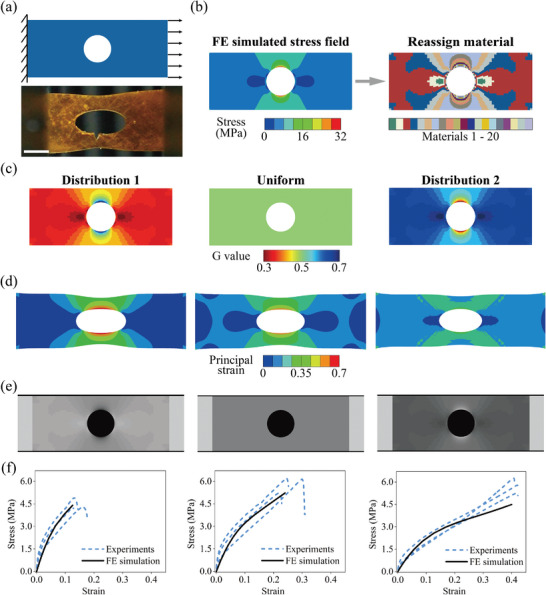
Reduced stress concentration via grayscale DLP printing. a) Experimental scheme and snapshot at break. b) FE‐simulated stress field and the corresponding material distribution. c) Three types of G distribution. i) Stiffer materials are assigned at places with smaller stress. ii) Uniform G = 0.5 for comparison. iii) Softer materials are assigned at places with smaller stress. d) FE‐simulated strain fields. e) Slice images of the plates. f) The comparison of experimental and FE‐simulated stress–strain curves of three structures (Scale bar: 5 mm).

When a plate with a central circular hole is subjected to longitudinal tensile stress, stress concentration occurs at two points on the diameter perpendicular to the long axis, which may lead to failures. High safety factors are generally needed to avoid the failure, which causes high material cost, and the weight and volume increases. Here, we show that the stress concentration in grayscale 3D‐printed structures can be reduced by rationally controlling the grayscale distributions. **Figure** **6**a shows the FE‐simulated and experimental results of a plate with a central circular hole under uniaxial tension. The size of the plate was 30 mm × 12 mm with a hole of 3 mm radius located at the center. A longitudinal displacement of 6 mm (20% strain) was applied. Stress concentration can be observed in the FE‐simulated stress field results, which leads to structural failure as the experimental result reveals (Figure 6a).

Grayscale DLP printing can reduce stress concentration by tuning the material distribution. In this work, the materials with different G were assigned according to the Mises stress field at 20% tensile strain, as shown in Figure 6b. Figure 6c demonstrates three different ways of modulus distributions using G. The area with smaller stress in FE simulation was allocated with a stiffer material in distribution 1 and a softer material in distribution 2. A uniform distribution was used for comparison. The detailed distribution of G is described below. All the elements of the plate were divided into 20 groups according to their Mises stress in Figure 6a. Similarly, G was divided into 20 groups from 0.3 to 0.7. We chose G varying from 0.3 to 0.7 because their failure strains were similar (around 65%). The divided G values were then assigned to the corresponding divided groups. In distribution 1, the element group with smaller stress was assigned with larger G (larger modulus), while it was assigned with a smaller G (smaller modulus) in distribution 2.

The FE‐simulated strain fields of the three structures under a uniaxial tensile strain of 20% are shown in Figure 6d. The maximum local strain in distribution 2 was around 42%, which was reduced by 27.6% compared to the structure with uniform distribution (with a maximum local strain ≈58%). In contrast, the maximum local strain distribution 1 was 70%, increased by around 21% compared to the structure with uniform distribution. The stress fields of three distribution are shown in Figure S9e. The maximum Mises stress of distribution 1 (30.8 MPa) was 3% larger than that of uniform distribution (30.0 MPa), while that of distribution 2 was reduced by 41.3% (17.6 MPa). As previously stated, the failure strain of the material was around 65%. Hence, the theoretical modeling predicts that distribution 1 fails, while distribution 2 remains intact.

Structures with three distributions were grayscale‐printed to validate the prediction from the theoretical model, with the slice images shown in Figure 6e. Uniaxial tensile tests were conducted. Three samples were printed and tested for each structure (Movie S2). Their corresponding stress–strain curves are shown in Figure 6f. The average failure strains of the three structures were around 13.2%, 24.2%, and 41.3%, respectively. By assigning a larger modulus at the spot with larger stress (distribution 2), the failure strain increased by 70.7%.

Note that the procedure used is not only increasing the grayscale value at the area of critical stress but also decreasing the grayscale value at the area with small stress, which makes the strain field more uniform using nonuniform grayscale value distribution. In this way, the area with small stress can withstand more strain, alleviating the strain at the original area with critical stress. We tested the sample with uniform G = 1 and nonuniform distribution based on its stress field, as shown in Figure S9a. Higher G was assigned for the region with larger stress and lower G for area of smaller stress. The FE‐simulated strain fields are shown in Figure S9b. The maximum principal strain of nonuniform G distribution was 50%, decreased by 16% compared to the distribution with uniform G = 1. The experimental snapshots are shown in Figure S9c and the stress–strain curves are shown in Figure S9d. The specimen with uniform G = 1 exhibits a large modulus but a small failure strain (13.6%). The nonuniform G distribution significantly increased the failure strain of the specimen (38.1%) and decreased the modulus.

### 3.2 Multimodal Soft Actuators and Manipulators

Controllable deformation is vital for soft robots to fulfill multifunctions but is still challenging because of the infinite degree of freedom and the nonlinear mechanical behaviors of the soft materials.^[^ ^49^, ^50^, ^51^ ^]^ Material anisotropy can be used to tune the deformation efficiently, but its development has been hindered by the limitations of current manufacturing methods. The developed design and fabrication framework provides a way to fabricate and design soft robots with material anisotropy. Here, we demonstrate two types of pneumatic soft actuators that can exhibit multimodal deformations with the same geometric parameters via designed G distributions: PneuNet and bellows soft actuators.

**Figure 7:**
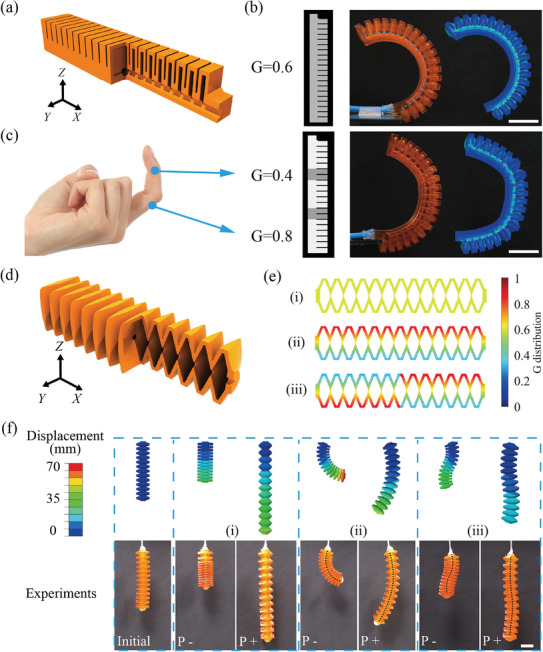
Multimodal grayscale‐printed pneumatic soft actuators. a) CAD model of a PneuNet soft actuator. b) Grayscale slice image, experiment, and FE simulation results of the PneuNet actuator with uniform G = 0.6. c) Human finger‐inspired PneuNet actuator with the custom G distribution. The deformed shapes obtained by experiments and FE simulation are compared. d) CAD model of a bellows soft actuator. (e) The G distributions of three bellows soft actuators: i) uniform distribution with G = 0.6, ii) graded distribution along the *Y*‐axis with G ranging from 0.4 to 0.8, iii) antisymmetric distribution with G varying from 0.4 to 0.8. f) FE simulation and experimental results of the bellows soft actuator with various G distributions and air pressures. “P +” refers to inflation and “P –” means deflation (Scale bar: 15 mm).

We first fabricated a PneuNet soft actuator with uniform G = 0.6. **Figure** **7**a shows the CAD model. The experimental and FE‐simulated deformed shapes are shown in Figure 7b under 70 kPa air pressure. Nearly constant curvatures were observed. In contrast, the human finger deformed with varying curvatures because of the phalanges and joint structures, enabling its conformal contact and load‐bearing properties simultaneously. Inspired by the human finger, we designed a PneuNet with varying curvatures using a nonuniform G distribution (Figure 7c). A higher G value (0.8) was used for the hard phalange part, while a lower G value (0.4) was used for the joints. The experiment snapshot and FE‐simulated deformed shape under 70 kPa are shown, which are similar to that of the human finger.

Next, bellows soft actuators were designed using varied G distributions. Bellows soft actuators generally exhibit elongation and contraction motions due to their symmetric geometry about the centerline. Versatile deformation modes can be formed by varying the G distributions. The CAD model of the bellows soft actuator is shown in Figure 7d. Here, we designed three different grayscale patterns (Figure 7e): i) uniform distribution with G = 0.6, ii) graded distribution along the *Y*‐axis with G ranging from 0.4 to 0.8, and iii) antisymmetric distribution with G varying from 0.4 to 0.8.

The FE‐simulated deformed shapes are shown in the upper row in Figure 7(f). Here, “P +” and “P –” refer to air inflation and deflation. The bellows soft actuators i) showed elongation and contraction when inflated and deflated. The soft actuator ii) contracted and bended to the right side when deflated, while it extended and bended to the left side when inflated. Interestingly, the pattern iii) soft actuator showed a contracted S‐shaped deformation when deflated and a reverse extended S‐shaped deformation when inflated, resulting from the antisymmetric G distribution. The three bellows soft actuators are grayscale‐printed and tested. The experiment snapshots are shown in the lower row of Figure 7f. Overall, the experimental results agree well with the FE predictions. The soft actuators showed finite deformations, e.g., actuator (i) exhibits an elongation of 46.7% and a contraction of 45%.

**Figure 8:**
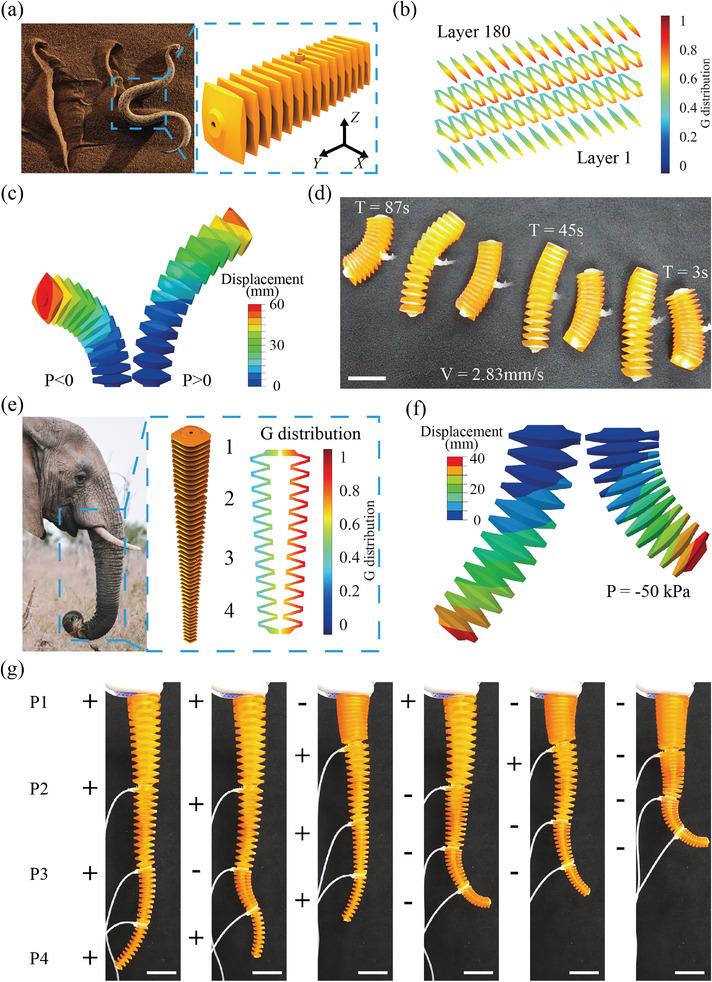
Bioinspired soft robots using grayscale‐printed bellows units. a) CAD model of the snake‐inspired soft robot. b) G distribution of the snake‐inspired soft robot (layers 1, 60,120,180). c) FE simulation of the snake‐inspired soft robot under inflation and deflation. d) Time‐lapse photo of the snake‐inspired soft robot showing sidewinding locomotion. e) CAD model and G distribution of the elephant trunk‐inspired soft manipulator. f) FE simulation of one section. g) Snapshots of the experiments showing various deformation modes (Scale bar: 30 mm).

Based on the theoretical prediction, we designed two bioinspired soft robots using grayscale DLP 3D‐printed bellows soft actuators. Inspired by the sidewinding locomotion of snakes on sand,^[^ ^52^ ^]^ we design a bellows soft robot (**Figure** **8**a and Movie S4) with gradients G values in both *X* and *Z* directions (Figure 8b). The *X*‐direction gradient leads to the in‐plane bending in different directions under inflation and deflation. Attributed to the *Z*‐direction gradient, the two ends of the soft robot bend out of the plane while the central part remains on the ground when deflated. When inflated, the central part was above the plane, and the two ends touched the ground (Figure 8c). The friction differences in these two modes lead to a sidewinding locomotion. The time‐lapse snapshot in Figure 8d shows the movement of the soft robot. The average velocity of the robot was ≈2.83 mm/s. The speed could be increased by applying a higher frequency or air pressure.

Inspired by the dexterous movements of the elephant trunk, we designed a multimodal soft manipulator by connecting four bellows soft actuators (Movie S4). A cone shape with a slope angle of 2° was used to improve the dexterity and reduce the gravity effect (Figure 8e). G distribution in the second section of the manipulator is shown. Other sections share a similar G pattern. The FE‐simulated deformation of Section 2 is shown in Figure 8f.

Four air inputs (P1, P2, P3, and P4) were introduced to control the four bellows soft actuators independently. The soft manipulators exhibited multimodal movements with different combinations of P1, P2, P3, and P4 (Figure 8g). The bellows soft actuators can realize multimodal movements using nonuniform G distribution while still maintaining the original symmetric geometry concerning its centerline. The symmetric geometry improves durability and prevents failure, compared to the asymmetric movements caused by geometric anisotropy of other bending soft actuators. Versatile deformation modes such as out‐of‐plane bending or swirling could be further accomplished by altering G distributions.

## 4 Discussions and Conclusion

Voxel 3D printing enables the precise control of material properties at the voxel level, which cannot be achieved using traditional manufacturing methods. Combining the geometrical design, the ability of voxel 3D printing to customize the material properties can be further enhanced, which is promising for developing functional structures in many fields such as soft robots,^[^ ^23^ ^]^ tissue engineering,^[^ ^53^ ^]^ 4D printing,^[^ ^54^ ^]^ and flexible electronics.^[^ ^55^ ^]^ The improvements in design freedom on material and geometric parameters significantly broaden the design space but further hinder both the prediction and inverse optimization of mechanical behaviors. Developing voxel design methods is still in its infancy currently. Combining the high‐resolution and support‐free voxel 3D printing technology, mathematics‐based modeling methods, and topological optimization may pave the way for the designing and application of voxel 3D printing.

Inspired by the dexterous motions of natural organism, we present a design and fabrication framework for grayscale DLP‐printed soft robots. A grayscale‐dependent hyperelastic Mooney–Rivlin constitutive model is proposed to characterize the nonlinear behavior of the materials with varying G. Voxel‐based FE simulations are used to predict the mechanical behaviors of the grayscale DLP 3D printed structures with arbitrarily distributed grayscale values. The printing parameters used for experiments should be consistent to ensure reliable prediction of the FE simulations. Altering these parameters introduces variations in the DoC distributions, leading to differences in material properties compared to the dogbone samples (Figure S11b). The proposed design and fabrication methods are then used to design structures, including structures with reduced stress concentration and multimodal soft robots.

Results show that: 1) G distribution can reduce the stress concentration of a plane with a central hole by assigning the G value according to the initial stress distribution, and increase the stretchability by ≈70%; 2) pneumatic soft actuators deformed with varying curvatures and complex trajectories can be realized by varying the G distributions; sidewinding soft robots and multimodal soft manipulators are designed based on the theoretical predictions. This work paves the way for integrated design and fabrication of functional structures using grayscale DLP 3D printing.

## 5 Experimental Section

### Hybrid Resin Preparation

The photocurable elastomer VeroClear (RGD810) was purchased from Stratasys (MN, USA) and chosen to be the base material. The monomer EAA (Ebecryl 113) was purchased from Allnex (Germany). The photo absorber Sudan I was purchased from Sigma‐Aldrich (MO, USA). All the materials mentioned above were used as received. The photocurable ink VEAA was prepared by mixing the VeroClear and EAA at the 4:1 weight ratio. The hybrid resin was then mixed thoroughly for 1 min at 2000 rpm, followed by centrifugation at 2200 rpm for 1 min, using the planetary centrifugal mixer (ARE‐310, Thinky, USA). 0.05% Sudan I was added to the mixture above at room temperature.

### Material Characterization

Uniaxial tensile tests were performed on a universal material testing machine (Instron 68SC‐2, USA) at room temperature with a test speed of 5 mm/min. Dogbone samples with different G = 0.1, 0.3, 0.5, 0.7, and 0.9 were printed. The UV projection time was fixed at 2 s. The dimensions of the specimen are shown in Figure S1(b). After the dogbone samples with different grayscale values were printed, they were rinsed with isopropyl alcohol, dried, and placed in the dark for 24 h to release the residual stress. Tensile tests were conducted on the 1^st^, 7^th^, and 14^th^ days after printing, as shown in Figure S14. It was observed that the material properties did not change much with time.

### A Self‐Built Grayscale DLP 3D Printing System

A self‐built bottom‐up DLP printing system was utilized to manufacture the printed structure. The system included the following parts: a linear translation stage LTS150 (ThorLabs, USA) to move the customized resin vat to the prescribed position for printing and a DLP projector 3DLP9000 (DLi, USA) to project a grayscale pattern to the bottom of the resin tank. The tank was coated with a PDMS membrane. The membrane was made by mixing the base material (Sylgard 184, Dow Corning) and the curing agent at a 10:1 weight ratio by the planetary centrifugal mixer (ARE‐310, Thinky, USA) at 2000 rpm for 1 min, followed by 1 min of centrifugation at 2200 rpm. The mixture was then poured down to the resin tank, degassed, and put in the heating oven at 50 °C for 10 h to completely solidify.

## Conflict of Interest

The authors declare no conflict of interest.

## Supporting information

Supporting Information

Supplemental Movie 1

Supplemental Movie 2

Supplemental Movie 3

Supplemental Movie 4

## Data Availability

The data that support the findings of this study are available from the corresponding author upon reasonable request.
