# Supplementary material for: Voxel Design of Grayscale DLP 3D‐Printed Soft Robots
Source: Adv Sci (Weinh). 2024 May 20;11(28):2309932. doi: 10.1002/advs.202309932 (PMC11267290; doi:10.1002/advs.202309932)
Supplement: Supplementary file 1 — Supporting Information [file ADVS-11-2309932-s003.pdf]

ADVANCED SCIENCE
Open Access

Supporting Information

for *Adv. Sci.*, DOI 10.1002/advs.202309932

Voxel Design of Grayscale DLP 3D-Printed Soft Robots

*Mengjie Zhang, Xiru Fan, Le Dong, Chengru Jiang, Oliver Weeger, Kun Zhou and Dong Wang**

Voxel design of grayscale DLP 3D-printed soft robots

*Mengjie Zhang, Xiru Fan, Le Dong, Chengru Jiang, Oliver Weeger, Kun Zhou, Dong Wang**

M. Zhang,X. Fan, L. Dong, C. Jiang, Prof. D. Wang
State Key Laboratory of Mechanical System and Vibration, School of Mechanical Engineering, Shanghai Jiao Tong University, Shanghai 200240, China, and Meta Robotics Institute, Shanghai Jiao Tong University, Shanghai 200240, China
Email Address: wang_dong@sjtu.edu.cn (D.W.)
Prof. Oliver Weeger
Cyber-Physical Simulation Group & Additive Manufacturing Center, Department of Mechanical Engineering, Technical University of Darmstadt, Dolivostr. 15, Darmstadt, 64293, Hessen, Germany
Prof. Kun Zhou
Singapore Centre for 3D Printing, School of Mechanical and Aerospace Engineering, Nanyang Technological University, Singapore 639798, Singapore

The PDF file includes:

S1 Validation of grayscale-dependent Mooney-Rivlin hyperelastic model

S2 Printing quality using grayscale values

S3 Light field simulation

S4 Mesh and voxel quality evaluation and sensitivity

S5 FE simulations

S6 Simulations and experiments on the plate with a circular hole.

S7 Triangular lattice metamaterial.

S8 Material system

Figure S1 Light intensities and dogbone experiments with various G.

Figure S2 Uniaxial compression tests for a cylinder and a functionally graded Kelvin foam.

Figure S3 The experimental and theoretically fitted stress-stretch ratio curves of grayscale-printed dogbone samples under different G from 0.1 to 1.

Figure S4 Enhancing printing quality using grayscale DLP printing.

Figure S5 Light field simulation of a prescribed grayscale distribution.

Figure S6 Slice images and experimental setups of cuboid structures.

Figure S7 Effects of mesh and voxel sizes.

Figure S8 Measured voxel side lengths of the grayscale-printed cuboid with patterns 1 to 4.

Figure S9 Simulations and experiments on the plate with a circular hole.

Figure S10 Programming stress-strain curves and Poisson's ratio of grayscale DLP 3D printed triangular lattice metamaterials.

Figure S11 Microstructure of the lattice metamaterial and the effect of the layer thickness.

Figure S12 Stress-strain curves of printed dogbone samples with different EAA concentrations and different grayscale values.

Figure S13 Stress-strain curves of the dogbone samples printed with different materials under various G.

Figure S14 Stress-strain curves of the printed dogbone samples with different G on the 1st, 7th, and 14th days after printing.

Table S1 Theoretically fitted C_{10}, C_{01}, and C_{11} and the corresponding adjusted R^2.

Table S2 Printing accuracy characterizations of square patterns.

Table S3 Comparison of the design and measured side length L of squares.

Other Supporting Information for this manuscript includes:

Movie S1 (.mp4 format). Uniaxial compression tests of the grayscale-printed functionally graded Kelvin foams.

Movie S2 (.mp4 format). Uniaxial tensile tests of the grayscale-printed plate with a central circular hole.

Movie S3 (.mp4 format). Uniaxial tensile tests of the grayscale-printed triangular lattice metamaterials.

Movie S4 (.mp4 format). Multimodal motions of grayscale-printed snake-inspired bellows soft robots and elephant trunk-inspired soft manipulators.

S1 Validation of grayscale-dependent Mooney-Rivlin hyperelastic model

Figure S1(a) shows the dependence of the light intensity on the grayscale values G. It can be seen that the light intensity is almost linearly increases with G. Figure S1(c) shows the stress-strain curves of the dogbone samples (CAD model shown in Figure S1(b)) printed with different G under uniaxial tensile tests. We can observe clearly that stress-strain curves are nonlinear. The dependences of Young's moduli E and fracture strain on G are obtained from the stress-strain curves and shown in Figure S1(d).

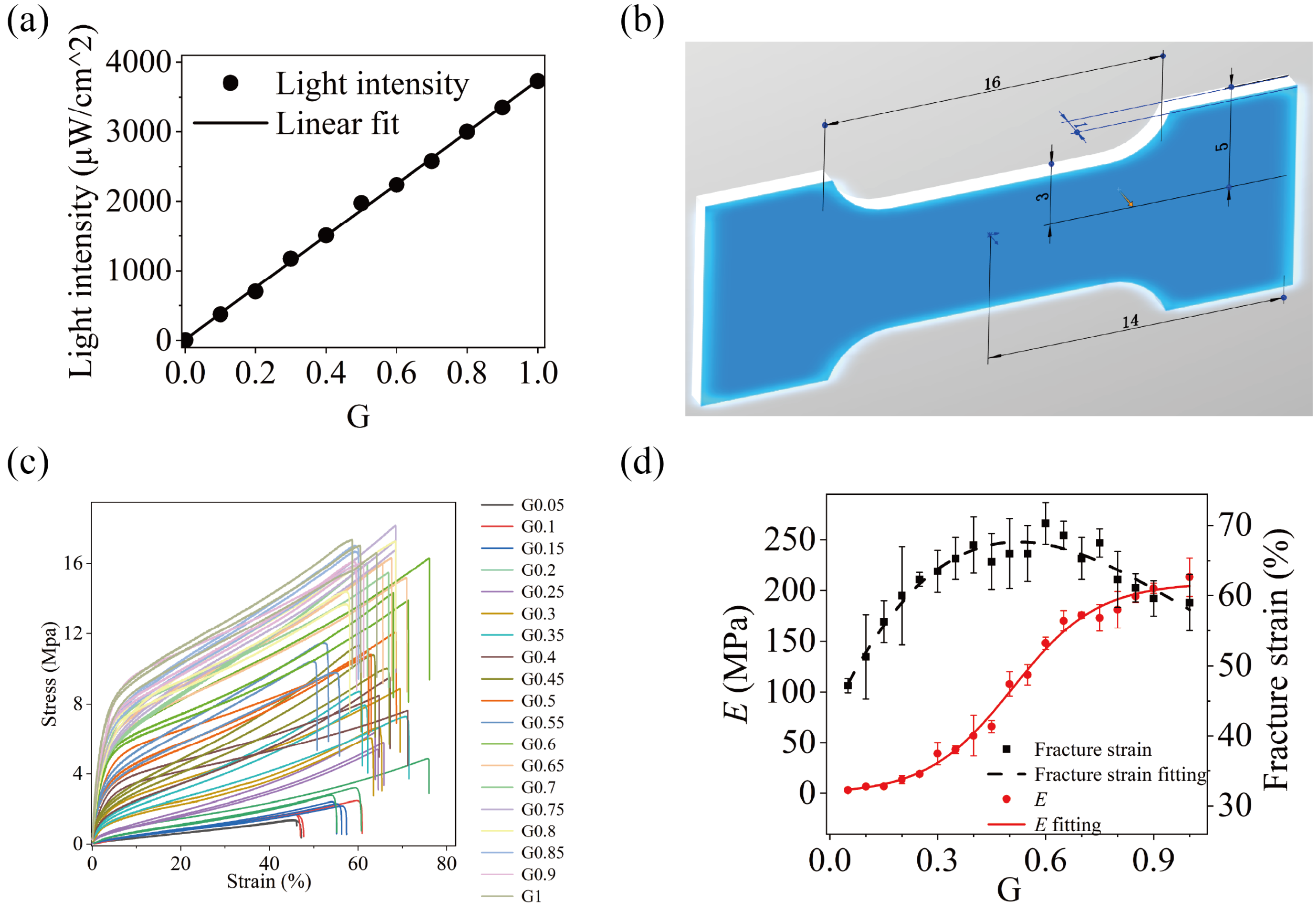

Figure S1. Light intensities and stress-strain curves with various G. (a) Scatter plot and linear fit of the dependence of the light intensity on G. (b) CAD model of the dogbone sample with dimensions (unit: mm). (c) Stress-strain curves of grayscale-printed dogbone samples with G ranging from 0.05 to 1. Three tests were conducted for each G.(d) The dependences of Young's moduli E and fracture strain on G.

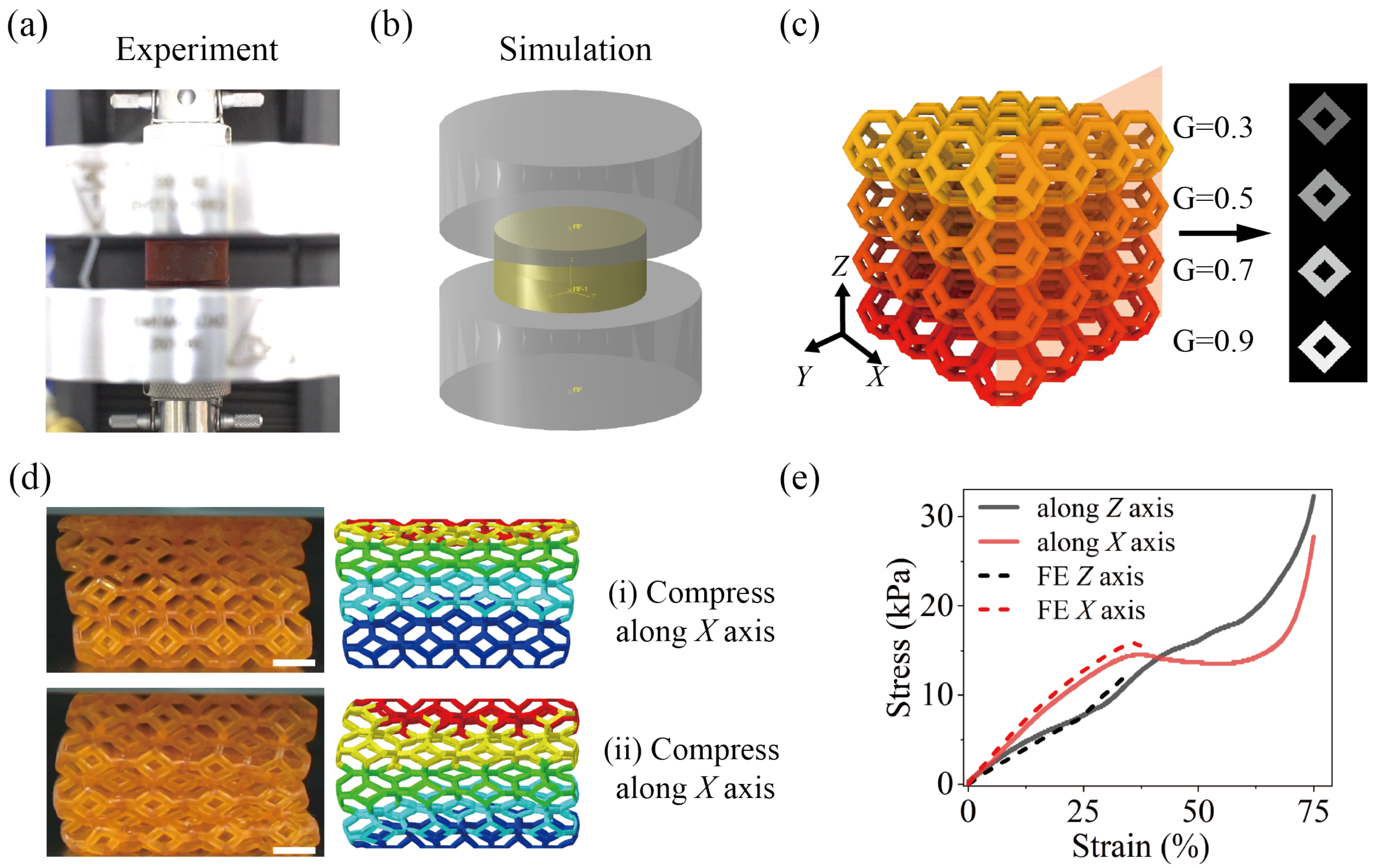

Figure S2. Uniaxial compression tests for cylinders and functionally graded Kelvin foam. (a) Experimental setup for a cylinder. (b) FE simulation setup for a cylinder. (c) CAD model and scheme of the G distribution of the functionally graded Kelvin foam. (d) Snapshots and FE simulation results of the functionall graded Kelvin foam under compression along the Z axis and X axis. (e) Stress-strain curves along the Z and X axes obtained from the uniaxial compression test and FE simulation.

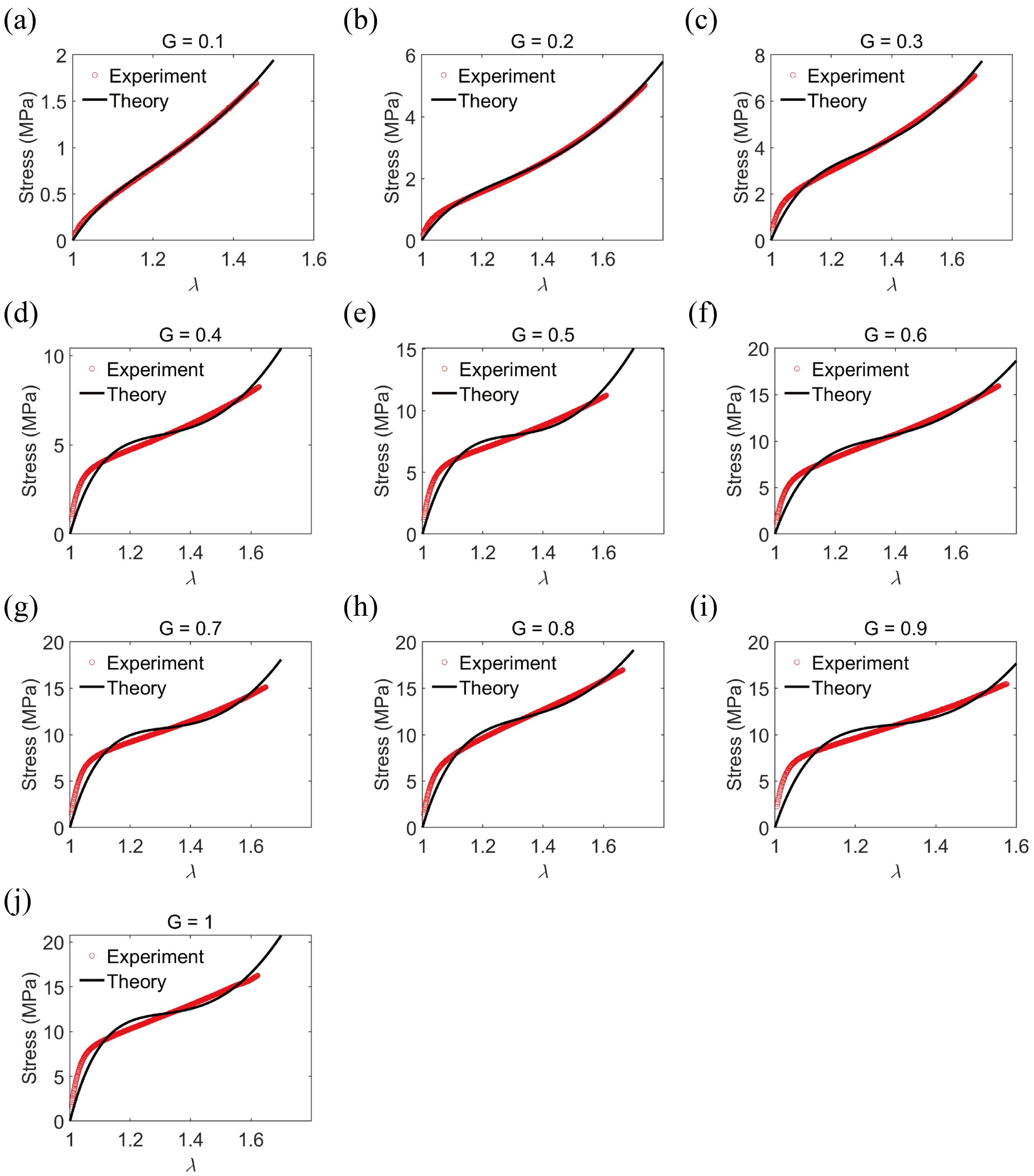

Figure S3. Experimental and theoretically fitted stress-stretch ratio curves of grayscale-printed dogbone samples under different G from 0.1 to 1.

Table S1: Theoretically fitted C_{10}, C_{01}, and C_{11} and the corresponding adjusted R^2 values.

G	C_{10}	C_{01}	C_{11}	Adjusted R^2
0.1	-1.666	2.772	0.928	0.999
0.2	-3.224	5.114	1.337	0.996
0.3	-11.053	15.734	3.428	0.986
0.4	-19.264	27.238	4.959	0.941
0.5	-28.602	34.852	8.040	0.922
0.6	-35.951	51.011	8.495	0.961
0.7	-46.796	64.734	11.780	0.926
0.8	-57.654	77.885	14.417	0.962
0.9	-68.153	91.179	16.892	0.911
1	-69.345	93.396	16.967	0.917

S2 Printing quality and accuracy

Grayscale values can also be used to tune the printing quality. When printing features occupy a large area, fracture may occur on a regular basis, possibly due to the large amount of heat produced in the photopolymerization. Take the Eiffel Tower model (Figure S4(a)) as an example. The main platform (Pattern I) has a large printing area, in which the structures often fail when a full light intensity is used (Figure S4(b)-i). By uniformly reducing the light intensity, this problem can be addressed (Figure S4(b)-ii).

However, reducing G may lead to the disappearance of the tiny features. In Pattern II where both tiny and large features exist, the tiny rails are not printed successfully (Figure S4(c)-iii). Differential G can then be used according to the size of the features, and the tiny rails and main platforms are printed simultaneously as shown in Figure S4(c)-iv. The ability of adjusting G according to the local features enables the high-precision 3D printing of multiscale structure, which is difficult for traditional DLP printing method.

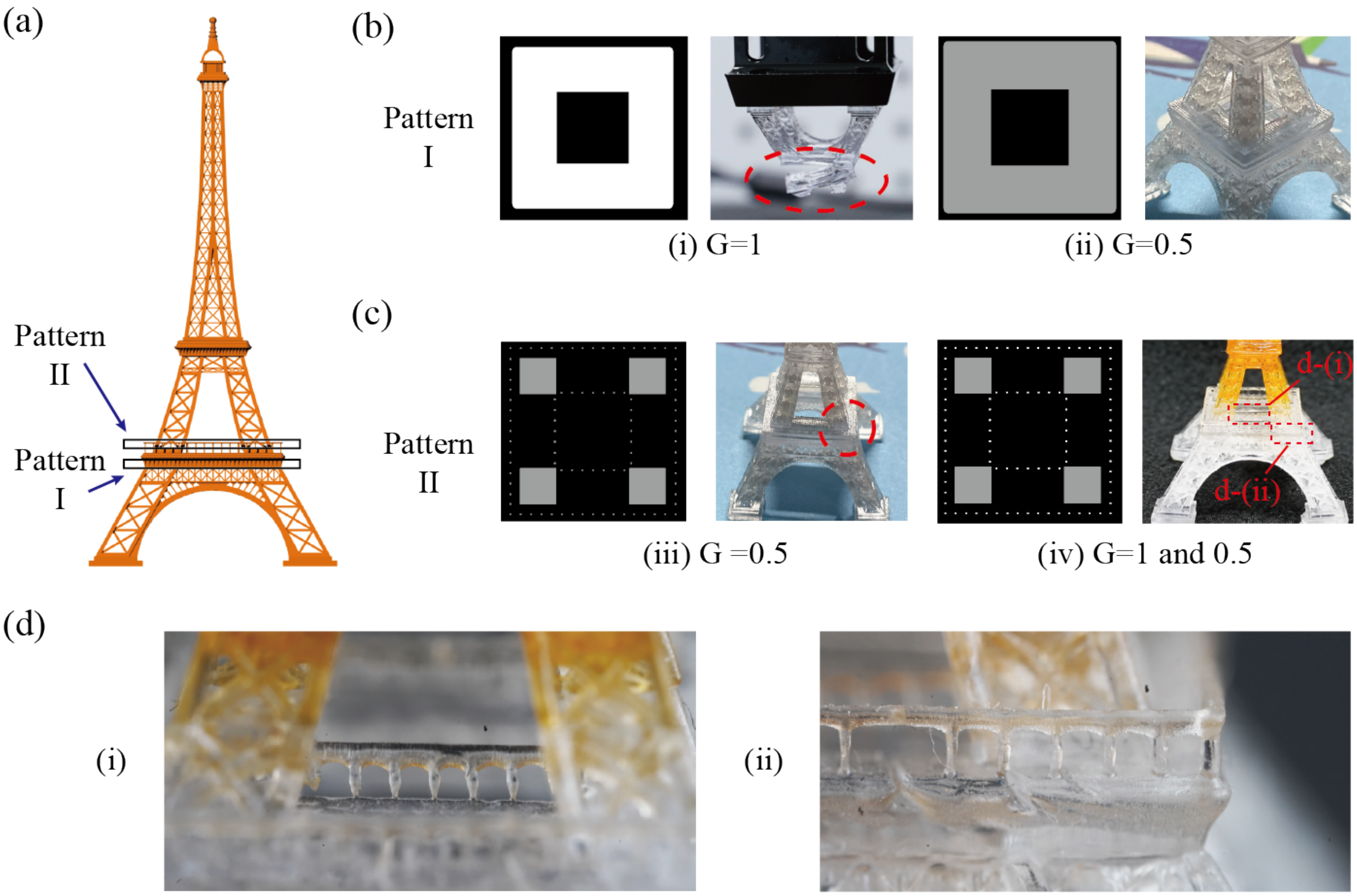

Figure S4. Enhancing the printing quality using grayscale DLP printing. (a) CAD model of the Eiffel tower with two boxes showing the place of the slicing pattern I and II. (b) Fracture generally occurred when G = 1 was used to print a large area (i). Successful printed using a uniform value of G = 0.5 (ii). (c) A small value of G may lead to the disappearance of small features (iii). Tiny and large features can be printed simultaneously using non-uniform grayscale distribution (iv). The enlarged pictures of the tiny structures marked by dashed boxes in (iv) are shown in (d).

Table S2: Printing accuracy characterizations of square patterns.

G	50 pixels (µm)	20 pixels (µm)	10 pixels (µm)	5 pixels (µm)	3 pixels (µm)	2 pixels (µm)
0.3	2036	831	424	202		
0.4	2049	841	419	208		
0.5	2058	842	427	214		
0.6	2067	855	429	215		
0.7	2085	847	432	217	82	
0.8	2090	854	436	219	170	
0.9	2107	847	441	215	162	88
Measured average	2070 ± 36	845 ± 12	432 ± 11	216 ± 9	138 ± 44	88
Designed value	2100	840	420	210	126	84
Maximum error	0.3%	1.8%	5.1%	4.3%	34.9%	4.8%

S3 Light field simulation

There is a gradient zone between regions with different G values. The light field of a prescribed grayscale distribution composed of four parts with different G is modeled. The grayscale pattern is shown in Figure S5(a). Gaussian beam distribution is assumed for the light projected from each pixel. Figure S5(b) shows the 3D plot of the normalized light intensity. We plot the light distribution on lines I1 and I2 in Figure S5(c). A gradient zone between regions with different G values can be observed. The gradient zone is defined as the interval between the valley and the peak of two adjacent waves with different G. The width of the gradient zone is estimated as ∼ 20µm.

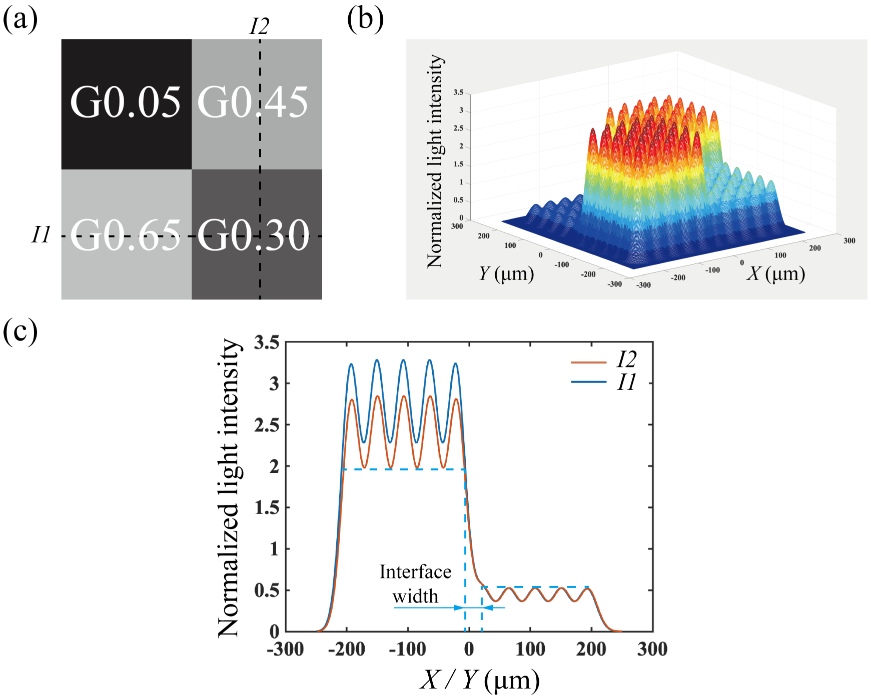

Figure S5. Light field simulation of a prescribed grayscale distribution. (a) Grayscale distribution is composed of four parts with different G. (b) 3D plot of the normalized light intensity of the prescribed grayscale distribution. (c) Normalized intensity along the $I1$ axis and $I2$ axis.

S4 Mesh and voxel quality evaluation and sensitivity

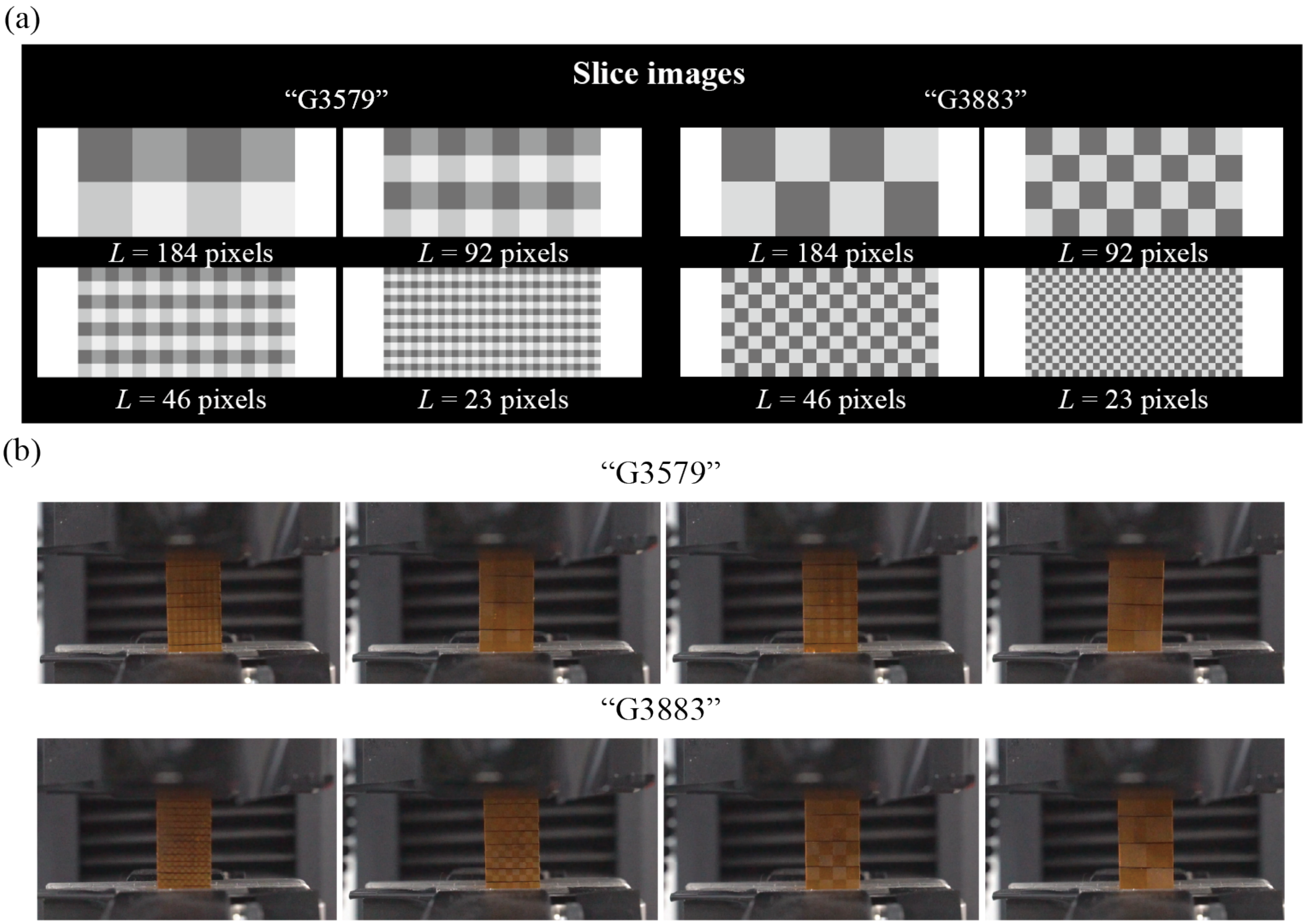

Figure S6. Slice images (a) and experimental setups (b) of cuboid structures.

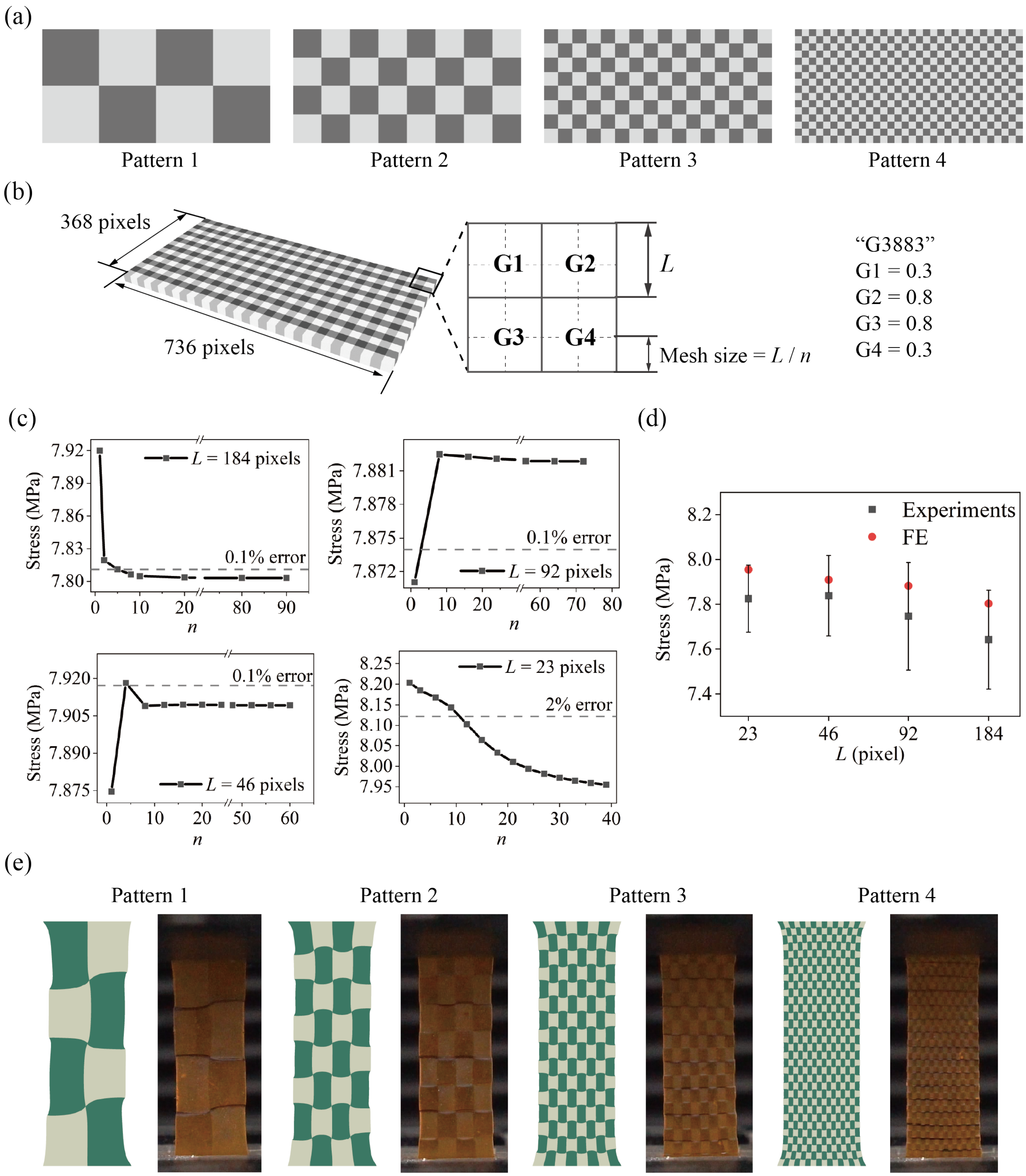

Figure S7. Effects of the and voxel sizes. (a) Four periodically arranged patterns. (b) Dimensions and grayscale value distributions (G3883) of the cuboid structure. (c) Mesh convergence analysis for grayscale-printed cuboid structure with four different patterns. (d) Quantitative comparisons between the experimental and converged FE-simulated stresses. (e) Comparison of the experimental and FE-simulated deformed shapes.

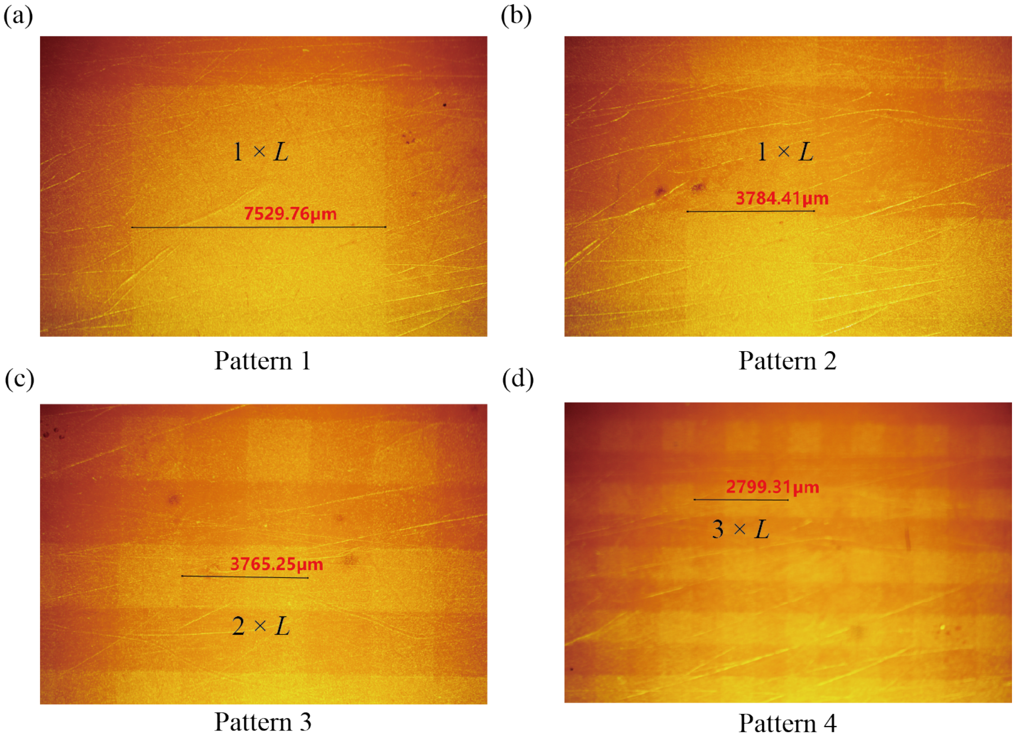

Figure S8. Measured voxel side lengths of the grayscale-printed cuboid with patterns 1 to 4.

Table S3: Comparison of the design and measured side length L of squares.

	Pattern 1	Pattern 2	Pattern 3	Pattern 4
Designed L	7.728 mm	3.864 mm	1.932 mm	0.966 mm
Measured L	7.530 mm	3.784 mm	1.882 mm	0.933 mm
Error	2.6%	2.1%	2.6%	3.4%

S5 FE simulations

All finite-element (FE) simulations are carried out through a commercially available FE software ABAQUS (SIMULIA, Providence, RI). Details of the FE simulations in Section 3 are described as follows.

In Section 3.1, 8-node biquadratic plane stress quadrilateral elements are used. A home-written python script is used to assign prescribed material constants to every element according to the stress field. Apart from the simulation in Section 3.2, all the models are analyzed using the general static step with nonlinear geometry in ABAQUS/Standard.

In Section S7, 3-node quadratic hybrid beam elements (B32H) are used. The boundary condition of one end is 22 mm (41.3% tensile strain). The structure is composed of the Mooney-Rivlin hyperelastic material, of which the material constants C_{10}(G), C_{01}(G), and C_{11}(G) are acquired in Section 3. Three microstructures in a single periodic unit are assigned with designed C_{10}(G), C_{01}(G), and C_{11}(G) using a home-written python script.

In Section 3.2, 10-node quadratic tetrahedron elements with hybrid formulation (C3D10H) are used for mesh. A uniform air pressure P is applied on the inner surface of the actuator. A home-written python script is used for cell partition and spatial material assignment.

S6 Simulations and experiments on the plate with a circular hole.

We tested the sample with uniform G = 1 and non-uniform distribution based on its stress field, as shown in Figure S9(a). Higher G is assigned for the larger stress region and lower G for the smaller stress area. The FE-simulated strain fields are shown in Figure S9(b). The maximum principal strain of non-uniform G distribution is 50%, decreased by 16% compared to the distribution with uniform G = 1. The experimental snapshots are shown in Figure S9(c) and the stress-strain curves are shown in Figure S9(d). The specimen with uniform G = 1 exhibits a large modulus but a small failure strain (13.6%). The non-uniform G distribution significantly increases the failure strain of the specimen (38.1%) and decreases the modulus. Figure S9(e) shows the FE-simulated strain fields of three G distribution.

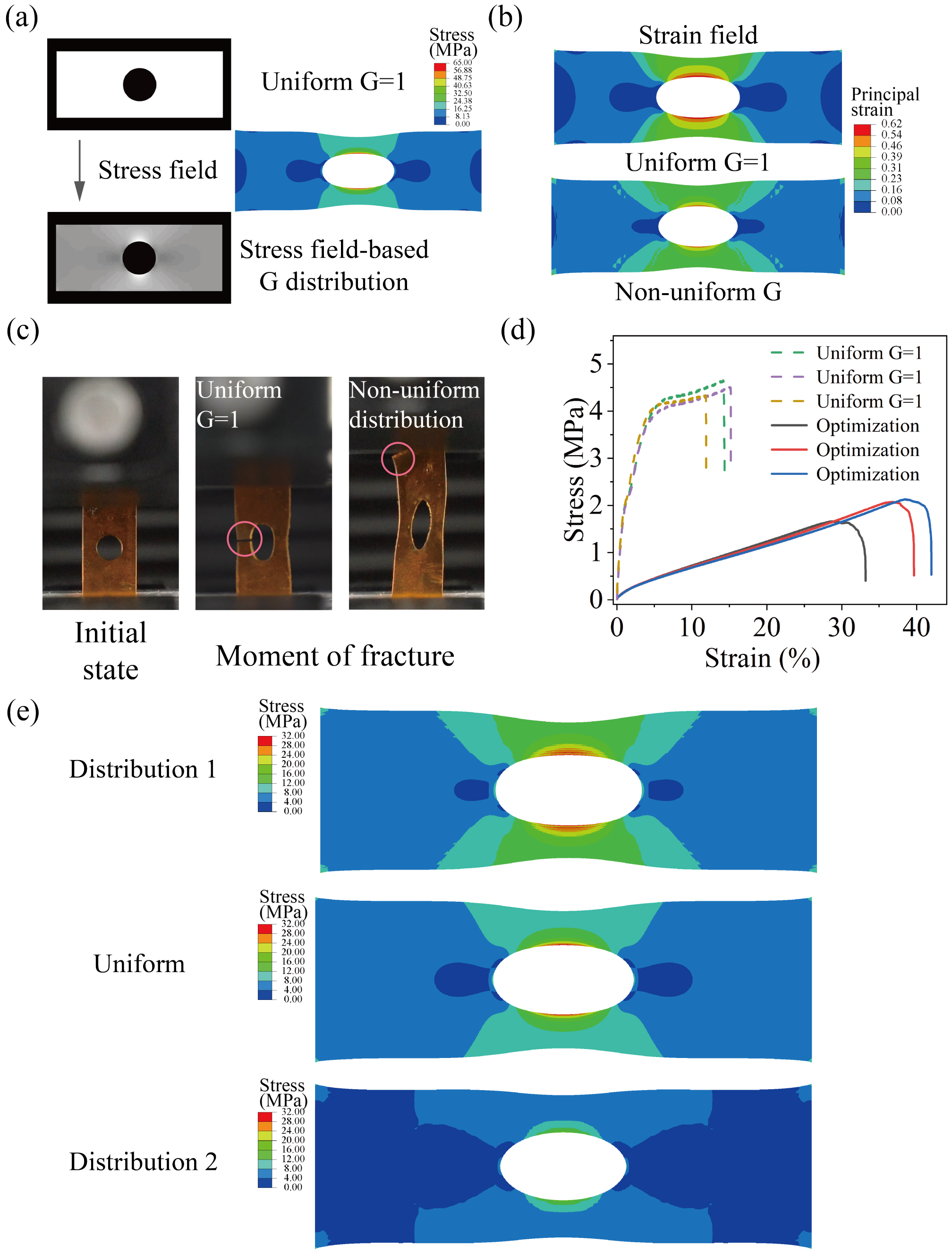

Figure S9. Simulations and experiments on the plate with a circular hole. (a) Grayscale distribution based on the stress field of the plate with uniform G=1. (b) FE-simulated strain field of the plate with uniform G = 1 and non-uniform G. The tensile strain is set at 20%. (c) Snapshots of the plates at the initial state and fracture. (d) Comparison of the experimental uniaxial stress-strain curves. (e) FE-simulated stress field of three distributions.

S7 Triangular lattice metamaterial.

Lattice metamaterials, referring to architected cellular materials with periodically arranged building blocks, exhibit extraordinary mechanical properties compared to their constituent materials.[1] Currently, their mechanical behaviors are generally tuned by geometric parameters and the effect of materials stiffness is neglected. Grayscale DLP 3D printing provides a way to control the mechanical properties using material properties distributions without changing the geometries. Here, we demonstrate that spatially controlled G can tune the mechanical behaviors of a triangular lattice metamaterial, including the stress-strain curves and Poisson's ratios.

A grayscale slice image of the printed triangular lattice metamaterial is shown in Figure S10(a). The representative unit of the triangular lattice is shown on the right side. G1, G2, and G3 represent the G of the unit cell's three microstructures, which can be directly controlled using the projected UV light. The left-upper half of the microstructures are generated by a curvature function $R(\alpha) = \alpha^2$, where R is the instantaneous radius of curvature and α is the slope angle (Figure S11(a)). Thus, the coordinates x and y of the microstructure are given by

$$x = \int_0^{\alpha} \alpha^2 \cos(\alpha) d\alpha, \tag{S1}$$

$$y = \int_0^{\alpha} \alpha^2 \sin(\alpha) d\alpha. \tag{S2}$$

The upper limit of the integral is α_{end}, which is given by the displacement boundary condition

$$y(\alpha_{end}) = 0. \tag{S3}$$

The right-bottom half is the antisymmetric shape with respect to the origin. The width and thickness of the microstructures are 0.5 mm and 1.5 mm, respectively. Four configurations with varying G1, G2, and G3 are shown in Figure S10(b). The configuration with G1 = 0.3, G2 = 0.5 and G3 = 0.7 is referred as "G357" (Figure S10(a)). The same principle also applies to the rest ("G555", "G735", "G573"). The lattice metamaterial is composed of 7×6 periodic units. The sliced images of other lattice metamaterials can be found in Figure S11(d).

FE-simulated stress-strain curves of the four lattice metamaterials under uniaxial tensile loading are plotted in Figure S10(c). All four lattice metamaterials exhibit J-shaped stress-strain curves. The J-shaped stress-strain curves result from the initial bending and uncurling and the sequential tension of the microstructure under loading. The lattice metamaterial with G357 exhibits the highest Young's modulus, while that with G573 has the lowest modulus. The moduli are comparable for lattice metamaterials with G555 and G735, indicating that the G of the third microstructure G3 is the dominant determinant of the modulus.

The FE-simulated Poisson's ratios are shown in Figure S10(d). The lattice metamaterials could exhibit positive, negative, or nearly zero Poisson's ratios. In particular, G555 exhibits nearly zero Poisson's ratio; G357 and G573 exhibit positive Poisson's ratio; and G735 exhibits negative Poisson's ratio. We can conclude that the Poisson's ratio depends on the grayscale of microstructures 1 (G1) and 2 (G2). If G1 is larger than G2 (for example, G735), the modulus of microstructure 1 is much larger than microstructure 2. Upon loading, microstructure 2 elongates and the deformation of microstructure 1 can be neglected. Therefore, the left node of the representative unit moves to the left-top direction to maintain geometric compatibility and the lattice metamaterials show a negative Poisson's ratio. In contrast, if G1 is smaller than G2 (for example, G357 and G573), microstructure 1 is compressed while microstructure 2 maintains its original length upon loading. The left node moves to the right-top direction and the lattice metamaterials show a positive Poisson's ratio. When G1 and G2 are the same (G555), the lattice metamaterials exhibit a nearly zero Poisson's ratio.

To validate the predicted mechanical behaviors, four lattice metamaterials are printed (Figure S11(c)) and tested under uniaxial load (Movie S3). The experimental stress-strain curves and Poisson's ratios are compared with the FE-simulated results in Figure S10 (c) and (d). The experimental and FE-simulated

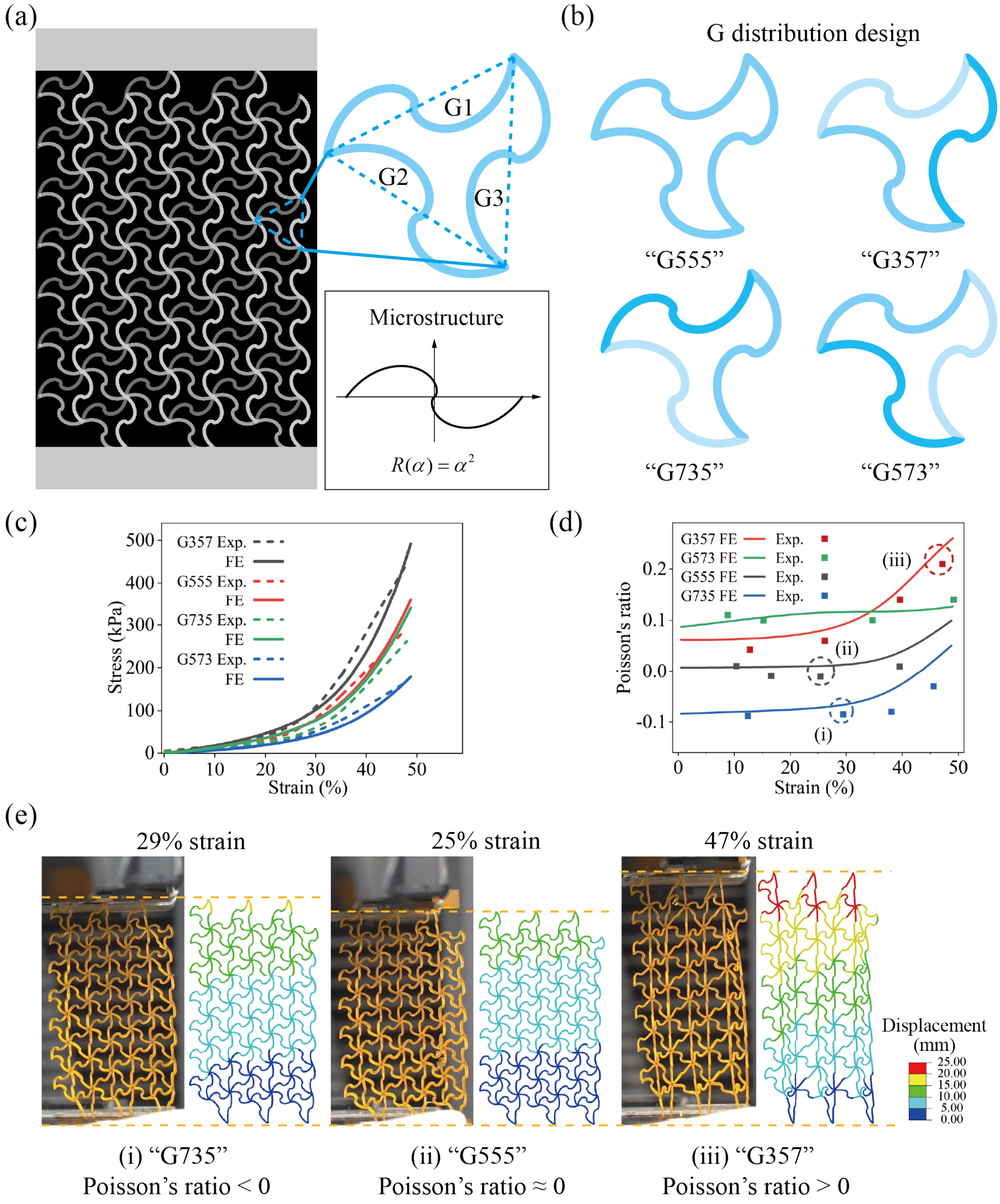

Figure S10. Programming stress-strain curves and Poisson's ratio of grayscale DLP 3D printed triangular lattice metamaterials. (a) A grayscale slice image of the printed triangular lattice metamaterials. Schematic illustrations of the triangular periodic unit and the curved microstructure are shown alongside. G1, G2, and G3 represent the G of the three microstructures. (b) Four G distributions of the periodic unit of the lattice metamaterials. The configuration with G1 = 0.3, G2 = 0.5, and G3 = 0.7 is referred to as "G357". The same definition also applies to the rest. (c) Experimental and FE-simulated stress-strain curves of four different G distributions under uniaxial tension. (d) FE-simulated Poisson's ratio-strain curves and experimental scatters. (e) Comparison of the experimental and FE-simulated deformed shapes of the designed structures under 29%, 25%, and 47% tensile strains. (Scale bar: 10 mm.)

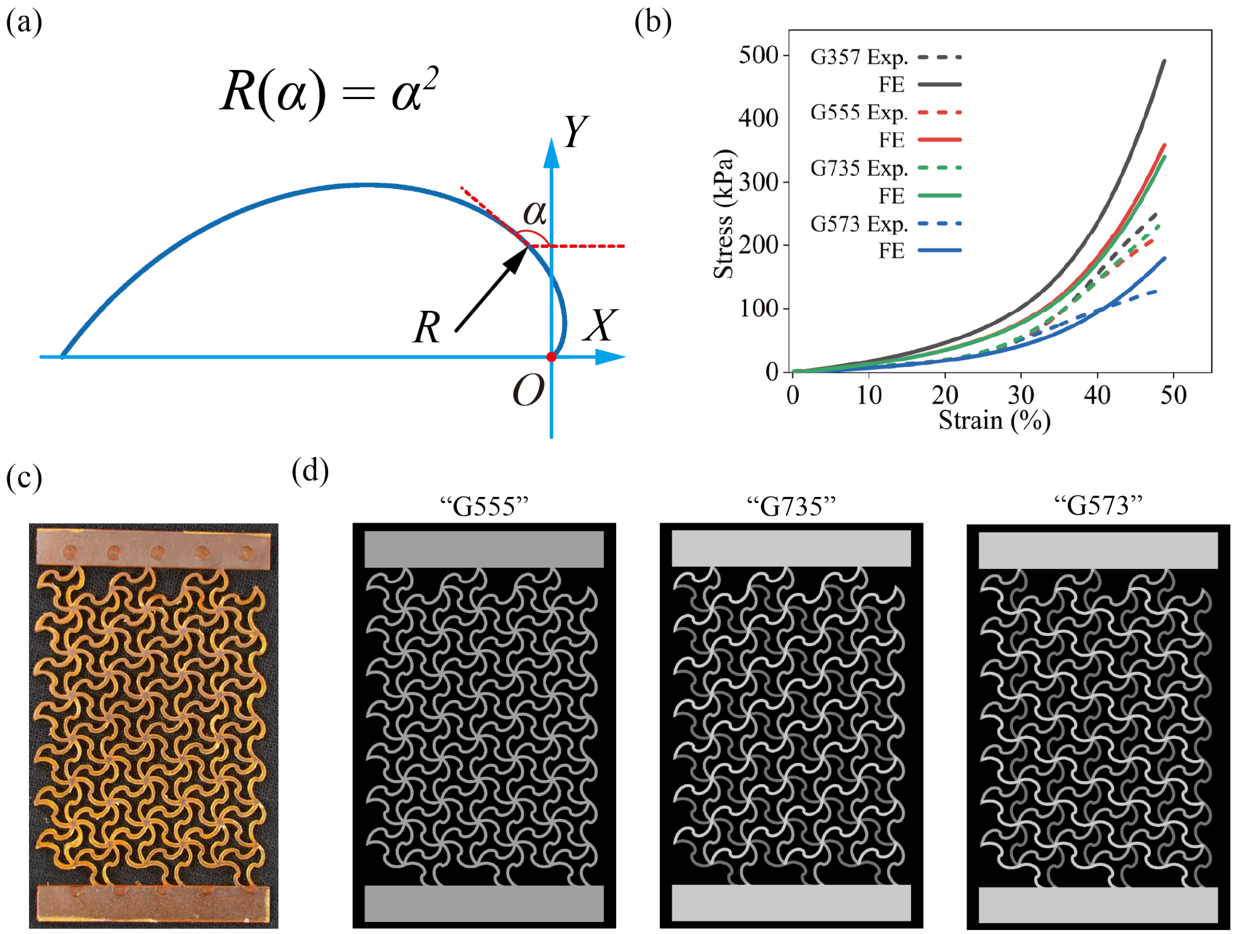

Figure S11. Microstructure of the lattice metamaterial and the effect of the layer thickness. (a) Schematic illustration of half of the microstructure. (b) FE-simulated stress-strain curves using layer thickness 50 µm. (c) The printed triangular lattice metamaterial. (d) Slice images of the triangular lattice metamaterial with different grayscale distributions "G555", "G735", and "G573".

deformed shapes for lattice metamaterials G735, G555, and G357 under 29%, 25%, and 47% tensile strain are shown in Figure S10 (e), respectively. The Poisson's ratios of the three shapes are marked in Figure S10 (d). The experimental and predicted mechanical behaviors agree well. All of the lattice metamaterials exhibit a large stretchability (larger than 45%).

Figure S11(b) compares the FE simulations and experimental results of four triangular lattice structures printed using a layer thickness of 50 μm, different from the usual layer thickness of 20 μm. Other printing parameters were kept unchanged. A significant discrepancy exists between the experimental results and FE simulations. The experimental curves are markedly lower than the FE simulations, attributed to the lower stiffness of materials printed with a 50 μm layer thickness compared to those printed with a 20 μm layer thickness, given the consistent use of the same light dose. Thus, the printing parameters used for experiments should be consistent to ensure reliable prediction of the FE simulations. Altering these parameters introduces variations in the degree of conversion distributions, leading to differences in material properties compared to the dogbone samples.

S8 Material system

S8.1 Different EAA concentrations

Dogbone samples with different Vero: EAA concentrations (20% EAA, 40% EAA, 70% EAA) are printed. 3 different grayscale values are used. Tensile tests were conducted, and the results are shown in Figure S12. We can observe that the moduli decrease from 203 MPa to 2.33 MPa when the EAA concentration increases from 20% to 70% at G = 1. At 20% EAA concentration, the failure strain does not change much with the grayscale value. However, the failure strain decreases significantly with G at 40% and 70% EAA concentration, which may cause failure in grayscale-printed soft robots. The decrease in the failure strain is partly due to the addition of EAA reducing the photo-initiator concentration in the VeroClear resin. The modulus and failure strain of pure VeroClear material is around 1.2 GPa and 20%, which is too stiff and easy to break in soft robot applications. Therefore, the VEAA material with 20% EAA is chosen as it exhibits a relatively larger fracture strain and a large variation of moduli.

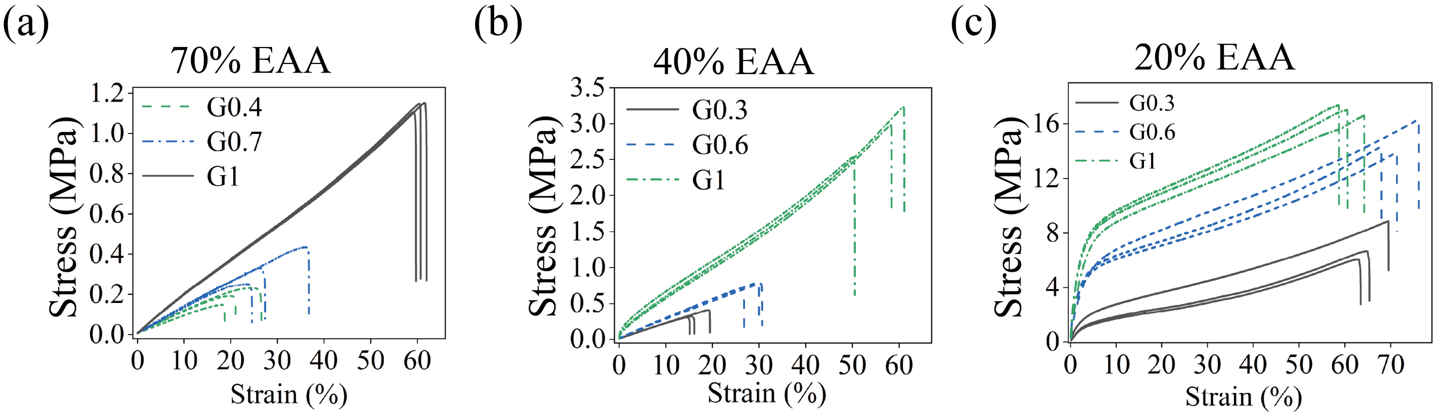

Figure S12. Stress-strain curves of printed dogbone samples with different EAA concentrations and different grayscale values.

S8.2 Different material

The proposed design and manufacturing framework is a general method. It can be used for other material systems as long as the mechanical properties of the photocurable resin depend on the light intensity. We measured the uniaxial tensile curves of dogbone samples printed using two other resins that were cured at various grayscale values (Figure S13). The first resin uses TangoPlus (FLX930) from Stratasys (MN, USA) and EAA with a mixing weight ratio of 6:4. The second resin mix VeroClear and TangoPlus with a weight ratio of 1:1. The results show that both material systems show grayscale-dependent mechanical

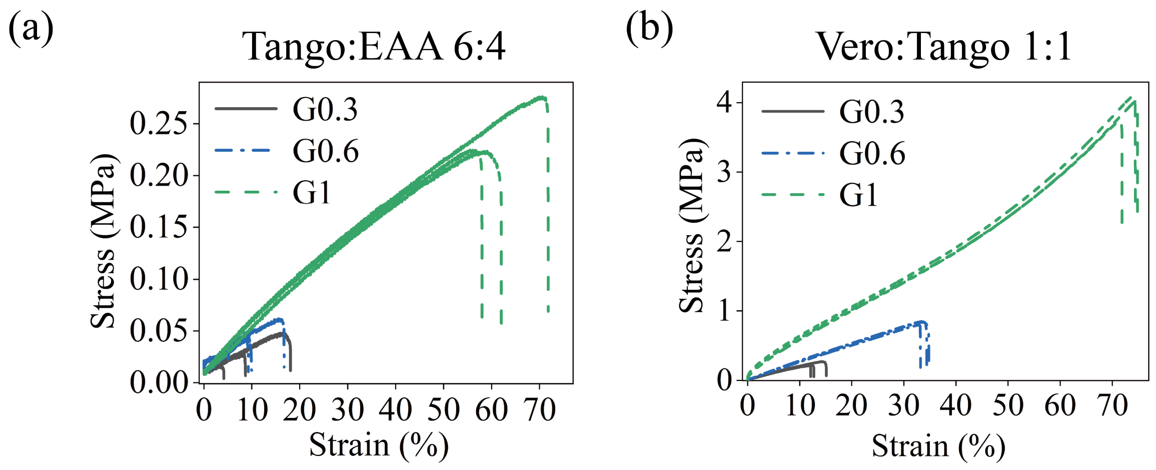

Figure S13. Stress-strain curves of the dogbone samples printed with different materials under various G. (a) Stress-strain curves of the dogbone samples printed with hybrid resin (TangoPlus: EAA = 6:4) under different G (0.3, 0.6, 1). (b) Stress-strain curves of the dogbone samples printed with hybrid resin (TangoPlus: VeroClear = 1: 1) under different G (0.3, 0.6, 1).

behaviors. Note that the constitutive model of the printed material should be chosen correctly to model the mechanical behavior more accurately. Once the material is changed, the constitutive model or material parameters should also be changed.

S8.3 grayscale-printed materials properties over time

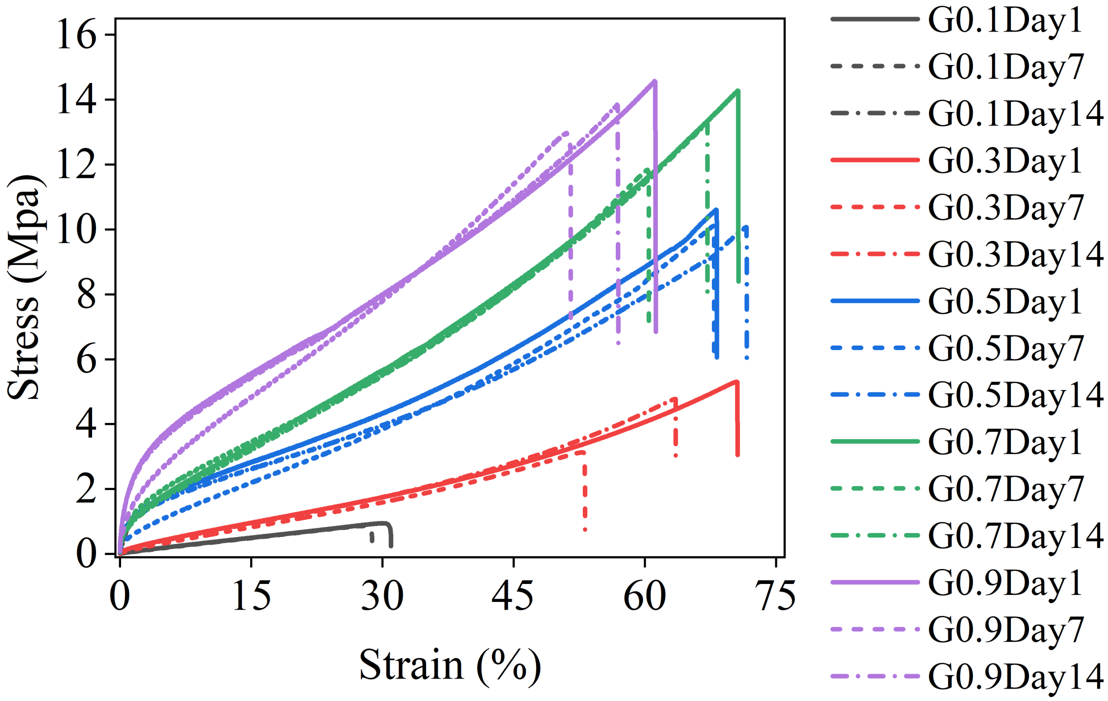

Figure S14. Stress-strain curves of the printed dog bone samples with different G on the 1st, 7th, and 14th days after printing.

References

[1] J. Chen, X. Liu, Y. Tian, W. Zhu, C. Yan, Y. Shi, L. B. Kong, H. J. Qi, K. Zhou, *Advanced Materials* **2022**, *34*, 5 2102877.
